# The HSV-1 Latency-Associated Transcript Functions to Repress Latent Phase Lytic Gene Expression and Suppress Virus Reactivation from Latently Infected Neurons

**DOI:** 10.1371/journal.ppat.1005539

**Published:** 2016-04-07

**Authors:** Michael P. Nicoll, William Hann, Maitreyi Shivkumar, Laura E. R. Harman, Viv Connor, Heather M. Coleman, João T. Proença, Stacey Efstathiou

**Affiliations:** Division of Virology, Department of Pathology, University of Cambridge, Cambridge, United Kingdom; Louisiana State University Health Sciences Center, UNITED STATES

## Abstract

Herpes simplex virus 1 (HSV-1) establishes life-long latent infection within sensory neurons, during which viral lytic gene expression is silenced. The only highly expressed viral gene product during latent infection is the latency-associated transcript (LAT), a non-protein coding RNA that has been strongly implicated in the epigenetic regulation of HSV-1 gene expression. We have investigated LAT-mediated control of latent gene expression using chromatin immunoprecipitation analyses and LAT-negative viruses engineered to express firefly luciferase or β-galactosidase from a heterologous lytic promoter. Whilst we were unable to determine a significant effect of LAT expression upon heterochromatin enrichment on latent HSV-1 genomes, we show that reporter gene expression from latent HSV-1 genomes occurs at a greater frequency in the absence of LAT. Furthermore, using luciferase reporter viruses we have observed that HSV-1 gene expression decreases during long-term latent infection, with a most marked effect during LAT-negative virus infection. Finally, using a fluorescent mouse model of infection to isolate and culture single latently infected neurons, we also show that reactivation occurs at a greater frequency from cultures harbouring LAT-negative HSV-1. Together, our data suggest that the HSV-1 LAT RNA represses HSV-1 gene expression in small populations of neurons within the mouse TG, a phenomenon that directly impacts upon the frequency of reactivation and the maintenance of the transcriptionally active latent reservoir.

## Author Summary

Like all herpesviruses, herpes simplex virus 1 (HSV-1) persistently infects an individual for their entire life. This persistent—or latent—virus is maintained as silenced DNA within the nuclei of sensory neurons, from which only the virus latency-associated transcript RNA is abundantly transcribed. Periodically, within an individual neuron, this silencing may be reversed and HSV-1 can reactivate to full virus replication. In this study we assess the role of the HSV-1 latency-associated transcript in the control of viral genome silencing and reactivation in mouse nervous tissue and individual neurons. We show that the latency-associated transcript decreases the expression of reporter genes engineered into the HSV-1 genome, as well as reducing the frequency of reactivation from individual neurons. Our study shows that in a proportion of latently-infected neurons, HSV-1 actively reduces the frequency of reactivation to full lytic replication. Such a function may increase the longevity of the infected neuron population within the infected individual, increasing the potential for life-long transmission to new hosts.

## Introduction

Herpes simplex viruses 1 (HSV-1) and 2 (HSV-2) are ancient human pathogens that are most commonly associated with sub-clinical and mild infections but can occasionally cause severe life threatening disease [1]. HSV-1 is most commonly associated with infection of the oral mucosa, and following productive primary infection at this site the virus is able to access the sensory neurons of the trigeminal ganglia (TG). Within these cells, HSV is able to establish a latent infection, characterised by a global reduction of lytic gene expression and an absence of infectious virus production. Periodically, latency is interrupted by reactivation of virion production from latent viral DNA, allowing for the transmission of the virus to new hosts. During latency, viral gene expression is largely restricted to the latency-associated transcript (LAT). The LAT is an 8.3kb primary transcript, which is spliced into stable 1.5 and 2 kb major LAT introns, as well as a 6.3 kb minor LAT exon that is processed into a number of microRNAs. The HSV LAT and its associated microRNA species appear to limit HSV immediate-early (IE) gene expression *in vitro* [2–4], as well as limiting the accumulation of viral lytic gene transcripts during acute and latent infection of mouse models [5, 6]. The LAT intron has also been strongly implicated in the global control of latent HSV gene expression in a number of studies describing the post-translational modification (PTM) of histones associated with viral promoters [7–11].

Using a reporter mouse model of infection, we have previously described a role for the LAT RNAs in maintaining the latently infected cell reservoir in the TG [12]. During infection with LAT-negative recombinants, latently infected neurons were lost at a rate of 1.7 neurons per TG per day. In contrast latent cell numbers remained stable up to 140 days post-infection with LAT-positive virus [12]. An additional study also identified a role for LAT in maintaining HSV reactivation competence following latent infection of the mouse TG [13]. Together, these reports suggest that the LAT RNAs enhance infected cell survival throughout latency and may do so by limiting reactivation of the virus from latency. In this study we have sought to determine whether LAT expression maintains the repression of the latent HSV genome *in vivo* by engineering a deletion of the LAT promoter into a previously characterised HSV-1 recombinant that expresses firefly luciferase [14]. With firefly luciferase under the control of the human cytomegalovirus (HCMV) major immediate early promoter (MIEP) (which is strongly expressed in neurons and whose activity does not require prior HSV lytic gene production), these viruses allow for a model of infection in which de-repression of the latent virus genome can be quantified directly from infected tissues. Using this approach to quantify both reporter gene expression and viral DNA loads within individual TGs, we have observed up to 4.9-fold greater median luciferase signal from LAT-negative mutants during latent infection in mouse TGs. We further identified that this increased gene expression is likely due to an increase in the number of cells in which virus gene expression occurs, and that reactivation frequency from individual latently infected neurons in *ex vivo* culture is also increased during LAT-negative HSV-1 infection. Finally, we demonstrate that in the absence of LAT expression, a significant and more prominent loss of luciferase signal relative to wildtype virus can be observed following long-term latent infection. Together, these data show that LAT represses gene expression from the virus genome as well as full productive reactivation.

## Results

### In vitro and in vivo characterisation of HSV-1 firefly luciferase recombinants with deletion and rescue of the LAT promoter

To investigate the role of LAT expression upon regulation of HSV-1 genome silencing, firefly luciferase-expressing viruses carrying deletions of the LAT promoter (LAP) were constructed on an HSV-1 strain SC16 genomic background, as described in Materials and Methods. Briefly, all viruses harbour an HCMV MIEP firefly luciferase expression cassette inserted in place of the viral U_S_5 gene. The independently generated LAT-deletion mutants harbour an HCMV MIEP GFP expression cassette (a 1369bp NsiI-PstI fragment from pEGFP-C1; Clontech) in place of the 203bp core LAT promoter, inserted in the opposite orientation to the LAT gene.

The rationale for these recombinants was to engineer a sensitive quantifiable reporter gene under the control of the HCMV MIEP into HSV-1, a promoter which has a) strong neuronal activity [15, 16], b) is suppressed during HSV-1 latency [15] and c) as a heterologous promoter is not dependent on HSV-1 gene products for its activation [17]. De-repression of gene expression from the HSV-1 genome should thus allow for strong expression of the reporter gene.

**Fig 1:**
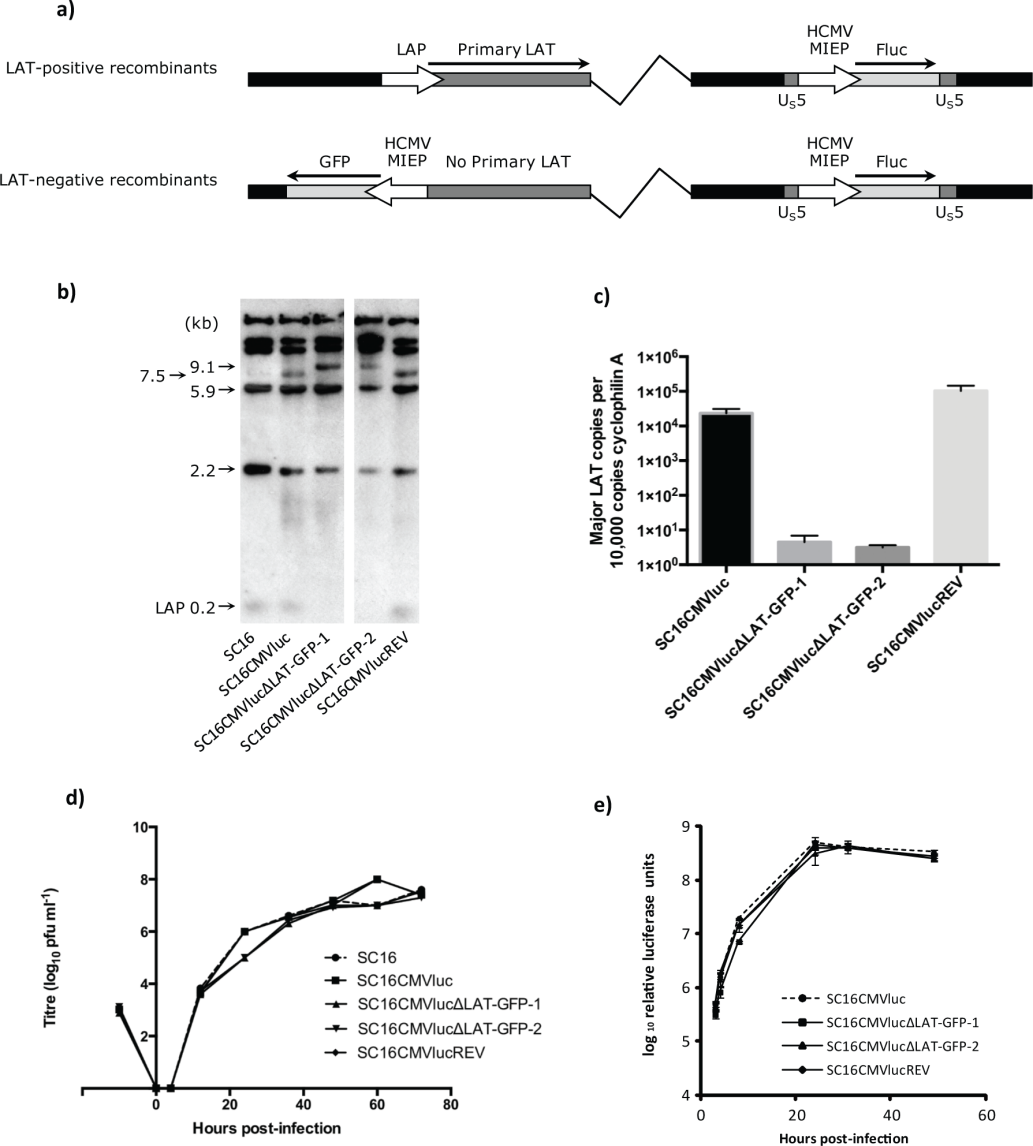
Generation of luciferase reporter viruses bearing deletions of the core LAT promoter. a) Genomic structures of SC16CMVluc and SC16CMVlucREV (LAT-positive recombinants), and SC16CMVlucΔLAT-GFP-1 and SC16CMVlucΔLAT-GFP-2 (LAT-negative recombinants). All four viruses harbour an HCMV MIEP-firefly luciferase expression cassette within the non-essential HSV-1 U_S_5 locus. b) Genomic structures as analyzed by Southern blot hybridization. Restriction digest with *Pst*I demonstrated all predicted restriction fragments, including the 203 bp LAT promoter. The 3.3-kb *Hpa*I fragment encoded within pPSTD1 was utilized as a probe. c) LAT expression was quantified by qRT-PCR from total TG cDNA. Primers for the major LAT intron and cyclophilin RNA were used. Histograms represent the mean (± SEM) numbers of major LAT RNA copies per 10^5^ copies of cyclophilin RNA from triplicate PCRs. d) Low (0.01) moi *in vitro* growth curves of recombinant viruses and wildtype strain performed in BHK cells. e) Firefly luciferase reporter viruses express equivalent levels of luciferase during infection *in vitro*. BHK cells were infected at an moi of 0.1 and harvested for luciferase assay at 3, 4, 8, 24, 31 and 49 hours post-infection. Symbols represent mean luciferase activity from three biological replicates. Error bars represent SEM.

The predicted genomic structures (Fig 1A) were confirmed by Southern blot hybridisation (Fig 1B).

**Fig 2:**
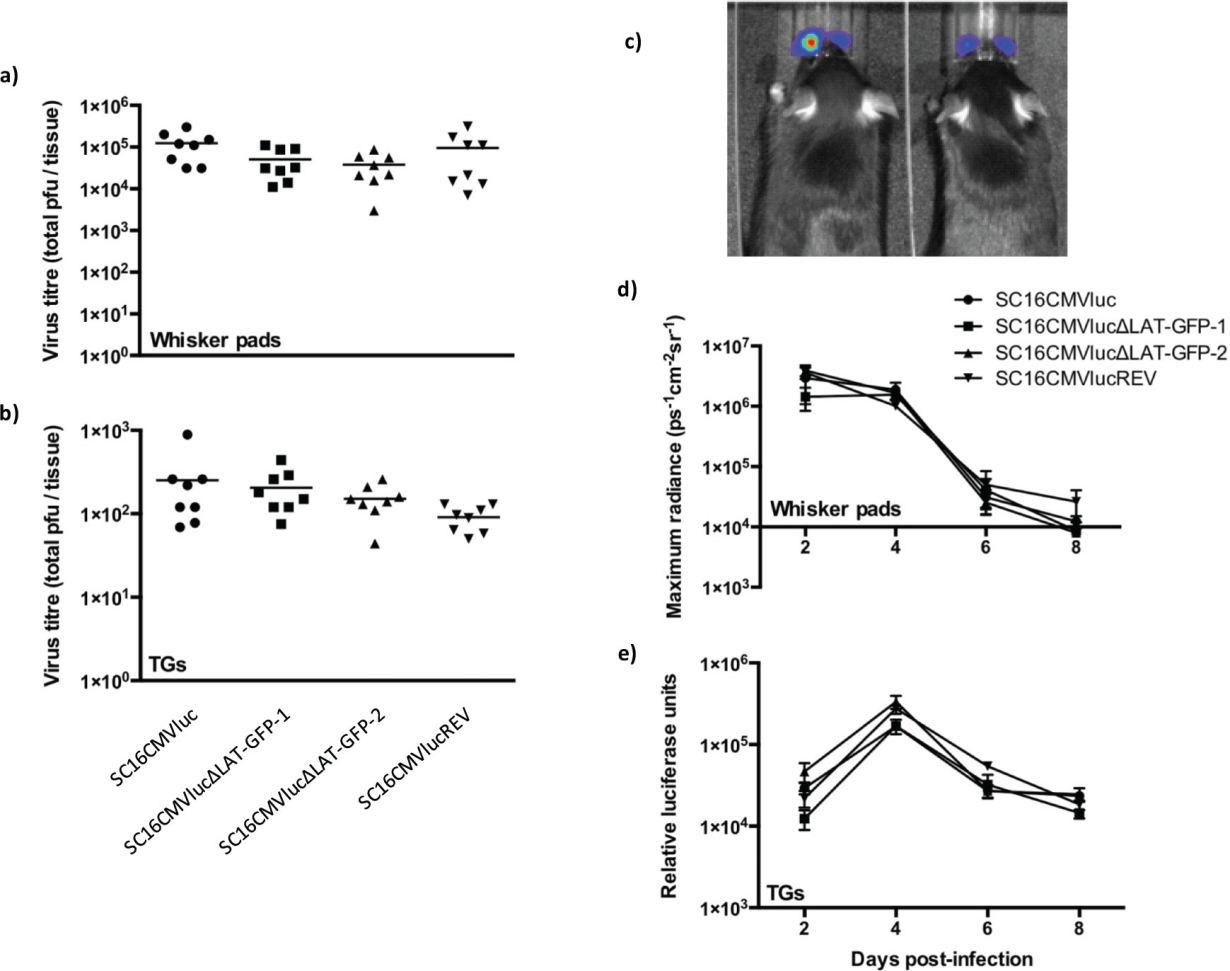
Luciferase reporter virus characterisation in vivo. a) Virus titres obtained from the whisker pads of C57BL/6, 4 dpi. Each symbol represents titres from an individual mouse and the floating bar represents the mean. b) Virus titres obtained from pairs of TG of C57BL/6, 4 dpi. Each symbol represents titres from an individual mouse and the floating bar represents the mean. c) Representative image of luciferase signal (colourimetric overlay) in live mice 2 days post-infection. d) Luciferase signal observed from reporter virus infection of C57BL/6 animals during acute infection. Each symbol represents mean luciferase signal (± SEM) (n = 4 mice 2 dpi, n = 7–8 on days 4–8 post infection). e) Luciferase signal observed from dissected TG homogenates during acute infection. Each symbol represents mean luciferase signal (± SEM) (n = 8 individual TGs).

Most notably, following *Pst*I digest the 203bp LAP DNA fragment was present in SC16CMVluc and SC16CMVlucREV but absent from independent SC16CMVlucΔLAT-GFP recombinants 1 and 2. Insertion of the HCMV-MIEP GFP expression cassette into the LAP locus was further confirmed by a shift from the 7.5 kb *Pst*I restriction DNA fragment observed in wildtype and revertant viruses to a 9.1 kb fragment in both independent LAT-negative viruses (Fig 1B). Quantitative reverse transcription PCR (qRT-PCR) analysis of RNA from TGs of mice latently infected with SC16CMVluc, SC16CMVlucREV and SC16CMVlucΔLAT-GFP recombinants 1 and 2 demonstrated a 5000-to-7500-fold reduction in major LAT expression from the LAT-negative mutants (Fig 1C), a similar or greater reduction in expression observed for LAT-deletion mutants described elsewhere [7, 10, 12]. All luciferase recombinants replicated with highly similar kinetics and in a manner indistinguishable to wildtype SC16 in a multi-step growth curve (Fig 1D). Furthermore, luciferase expression was equivalent from all viruses during infection in cell culture (Fig 1E). To assess virus replication *in vivo*, C57BL/6 mice were infected with SC16CMVluc, SC16CMVlucREV and SC16CMVlucΔLAT-GFP recombinants 1 and 2 (as detailed in Materials and Methods) and infectious virus titres were determined for whisker pad and TG tissues four days post-infection (d.p.i.). By comparison with the previously characterised SC16CMVluc recombinant [14] average titres were similar between all recombinants in both the whisker pads and TGs (Fig 2A and 2B).

No significant difference in virus titres was ascertained from the whisker pads or TGs (P = 0.1 and 0.2, respectively; one-way analysis of variance, n = 8 mice per virus). To further scrutinise virus kinetics *in vivo*, mice were inoculated as before but assessed by live luciferase imaging (Fig 2C), which allows for repeated analysis of the same mice throughout infection. Infection kinetics of all four viruses was highly similar in the mouse whisker pads (Fig 2D). In an independent experiment, similar luciferase expression kinetics were also observed in the dissected TG of mice during acute infection with each virus, using a 96-well plate luminometer (Fig 2E).

### In the absence of LAT transcription, HSV-1 recombinants express elevated levels of luciferase during neuronal latency

We have previously reported that LAT expression leads to stable maintenance of infected cell populations throughout long-term latent infection [12], indicating that the LAT RNAs facilitate survival of latently infected neurons over protracted time periods. One explanation for this phenomenon could be an increased frequency of lytic gene expression during latency (potentially proceeding to infectious virus production), followed by cell death.

**Fig 3:**
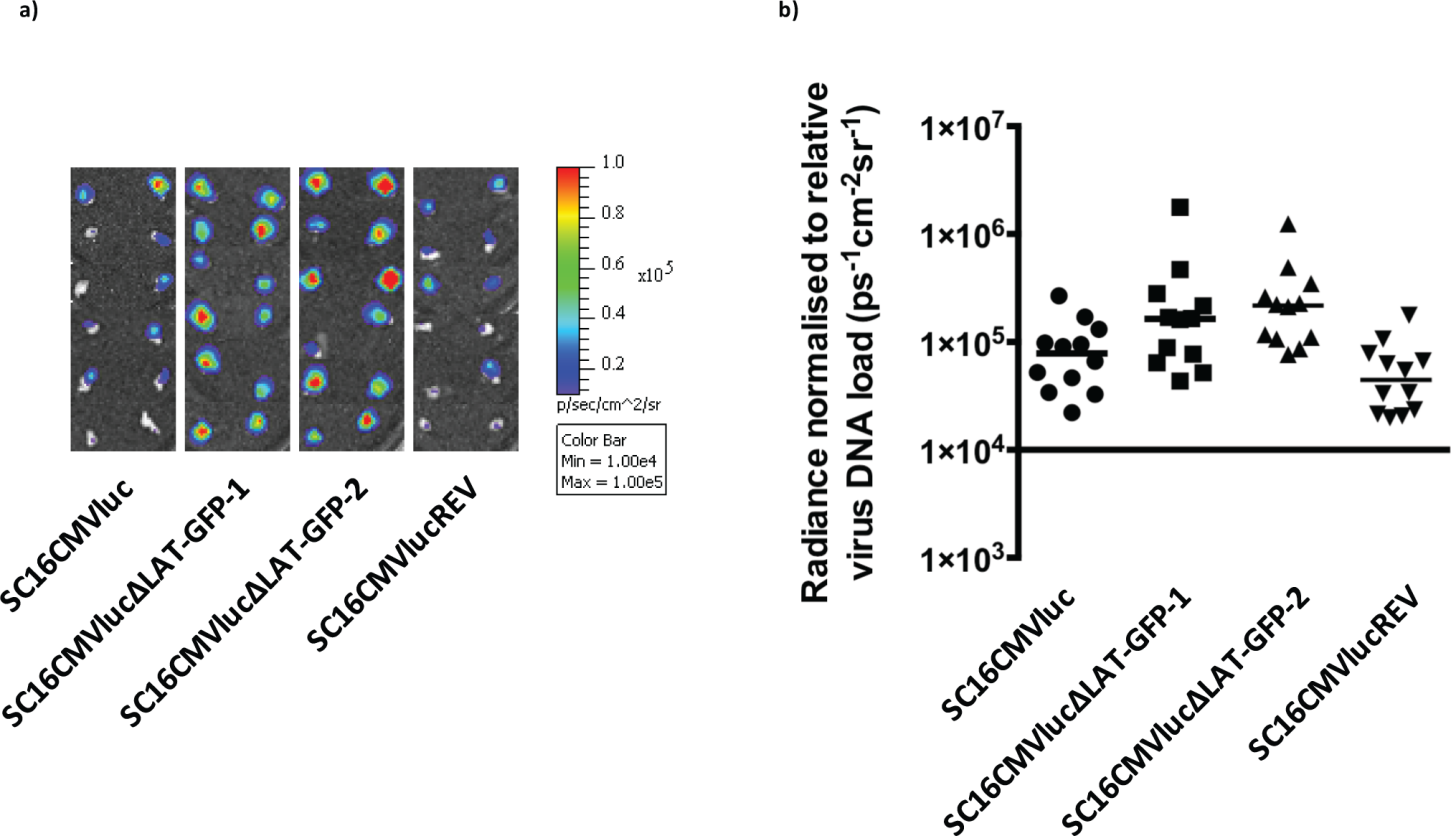
Luciferase expression is higher during LAT-negative HSV-1 infection in latent trigeminal ganglia. a) Following injection of D-luciferin substrate, TGs were dissected at set times from each mouse and luciferase intensity was measured. Displayed are composite photographs of dissected TGs with luciferase signal represented by a colourimetric overlay. The luciferase signal overlay is relative to the displayed scale. b) Luciferase signal was quantified with Living Image software and normalised to relative HSV-1 DNA loads within the same TGs. Each symbol represents normalised luciferase signal from an individual TG. Floating bars represent the median signal of each virus group. A signal of 1x10^4^ ps^-1^cm^-2^sr^-1^ represents a background threshold of detection. Statistically significant differences were observed between LAT-positive and LAT-negative viruses, with the exception of SC16CMVlucΔLAT-GFP-1 vs SC16CMVluc (SC16CMVlucΔLAT-GFP-1: P = 0.09 and 0.007 compared to SC16CMVluc and SC16CMVlucREV, respectively; SC16CMVlucΔLAT-GFP-2: P = 0.04 and 0.0002 compared to SC16CMVluc and SC16CMVlucREV, respectively; Kruskal-Wallis with Mann-Whitney post-tests).

To observe the earliest stages of virus reactivation (measured by CMV promoter de-repression and induction of luciferase gene expression from the latent HSV genome) we infected 24 C57BL/6 mice with SC16CMVluc, SC16CMVlucREV and SC16CMVlucΔLAT-GFP recombinants 1 and 2 for TG luciferase imaging. This methodology allows for the direct measurement of luciferase activity and thus de-repression of latent virus genomes within intact TGs dissected 30 days p.i (Fig 3A). Following capture of luciferase signal, total DNA was extracted from each individual TG in order to normalise signal intensity to virus loads (Fig 3B). A statistically significant increase in luciferase signal was observed following establishment of latency with both LAT-negative mutants, which was 2.1–4.9-fold higher relative to the wildtype and revertant recombinants (Fig 3B). Similar luciferase signals were recorded from both LAT-negative virus infections, and SC16CMVlucREV possessed an insignificant yet slightly lower median signal in comparison to SC16CMVluc (P = 0.34 and 0.17, respectively; Kruskal-Wallis with Mann-Whitney post-tests).

The elevation in luciferase signal from LAT-negative viruses was corroborated in a repeat experiment measuring TG pairs (S1 Fig). Together these data demonstrate enhanced expression of an exogenous reporter gene in the absence of LAT expression, consistent with a relaxation of global gene silencing during latency.

### Chromatin immunoprecipitation analysis of the latent HSV-1 genome

**Fig 4:**
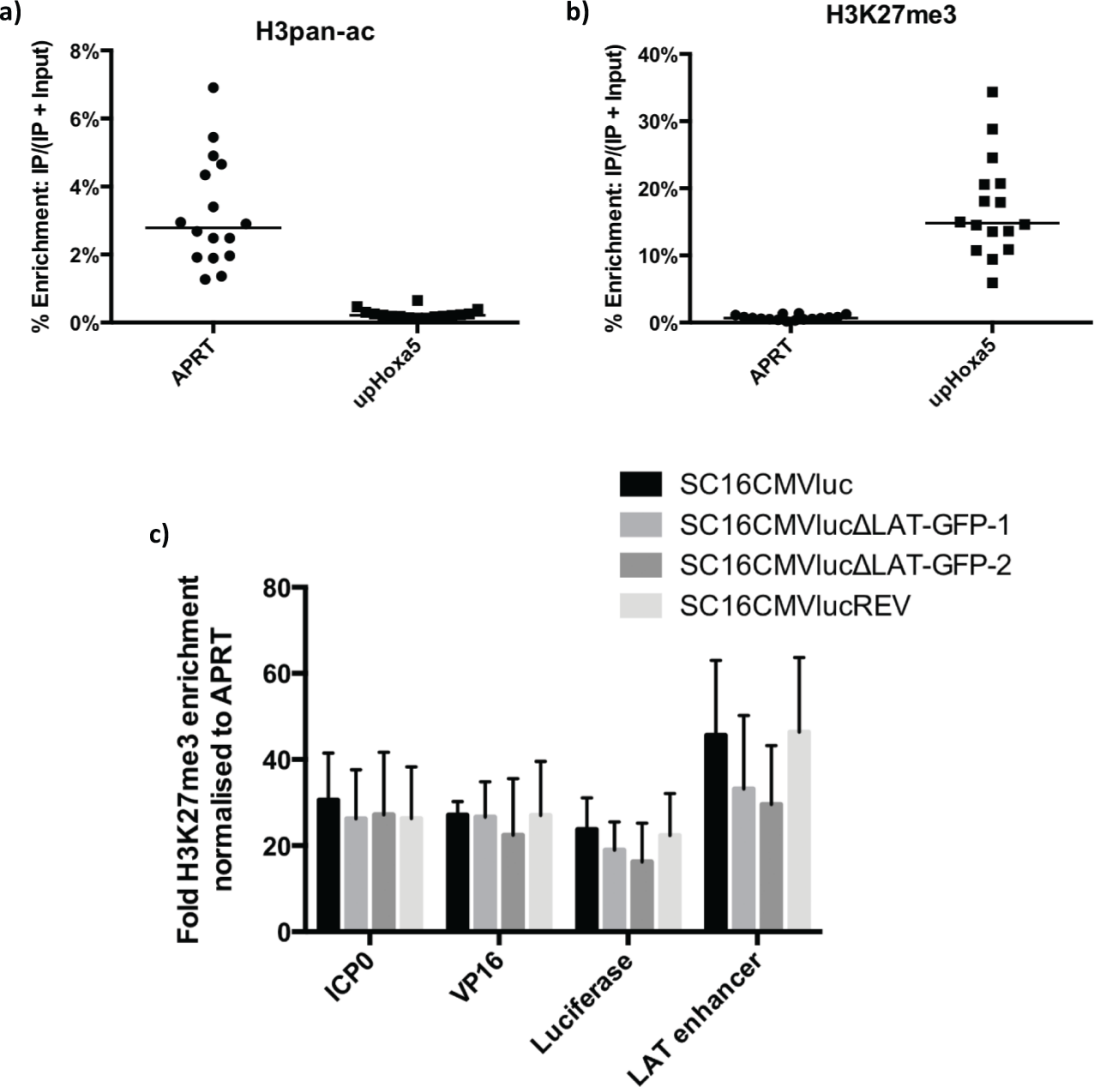
Latent HSV-1 genomes are highly enriched in facultative heterochromatin. Chromatin aliquots isolated from latently infected mouse TG were precipitated with antibodies to H3ac or H3K27me3. The specificity of each antibody was assessed by quantifying the relative enrichment of sequences known to be associated with euchromatin (APRT) or facultative heterochromatin (up*Hoxa5*). Symbols represent percentage enrichment of sequences from individual TGs (n = 16) and floating bars represent the means of these data following immunoprecipitation with a) anti-H3ac and b) anti-H3K27me3 antibodies. c) Fold enrichment of four virus sequences with facultative heterochromatin marker H3K27me3. Histogram displays the average of four independent experiments (+ SEM), each comprising groups of three pooled TGs per virus. Fold enrichment of H3K27me3 immunoprecipitation (IP) was calculated as: [Virus sequence IP ÷ (IP + Input)] ÷ [cellular APRT IP ÷ (IP + Input)].

Previous investigations have strongly indicated a role for LAT expression (and the major LAT intron, in particular) in the epigenetic regulation of HSV-1 gene expression [7–10]. In order to examine the contribution of chromatin regulation on the observed luciferase expression phenotype in our experiments, as well as assessing the level of epigenetic repression upon endogenous HSV-1 promoters, we next sought to determine the enrichment of both activating and repressive modified histones by chromatin immunoprecipitation analysis. To investigate this, we utilised polyclonal antibodies specific to pan-acetylated histone protein 3 (H3ac: a marker of activating euchromatin) and trimethylated lysine 27 of histone protein 3 (H3K27me3: a marker of repressive facultative heterochromatin). We confirmed the specificity of these antibodies within mouse TGs by specifically immunoprecipitating known euchromatic and heterochromatin regions of the mouse genome [adenine phosphoribosyltransferase: APRT and an upstream region of gene *hoxa*5 [8]] with H3ac and H3K27me3 antibodies, respectively (Fig 4A and 4B).

Forty-eight C57BL/6 mice were infected with 10^6^ pfu of SC16CMVluc, SC16CMVlucΔLAT-GFP-1, SC16CMVlucΔLAT-GFP-2 or SC16CMVlucREV. Chromatin was isolated 30 dpi from the TGs of three pooled mice per virus, as described in Materials and Methods. Four biological repeats per virus were conducted in total. To assess the global regulation of the HSV-1 genome we measured histone enrichment upon the endogenous VP16 and ICP0 promoters, as well as a region of the LAT enhancer. As three HCMV MIEP promoters are present in both LAT-negative viruses, we assessed chromatin enrichment upon the very 5' coding sequence of the firefly luciferase cassette. We observed negligible levels of pan-acetylated H3 enrichment on these sequences, with all consistently less enriched than the facultative heterochromatin control sequence of up*hoxa5* (S2A Fig). We also failed to detect any immunoprecipitated H3ac in a number of PCRs (S1 Dataset). A notable enrichment of H3ac was detected on the LAT enhancer in only one of four biological repeats from a single virus (S2B Fig). In contrast, reciprocal analysis with H3K27me3 demonstrated that all four target regions were highly enriched with facultative heterochromatin (Fig 4C). These data confirm that the vast majority of HSV-1 genomes are indeed highly repressed during latency. More surprisingly, we were unable to determine a statistically significant difference in H3K27me3 enrichment between LAT-positive and LAT-negative viruses at any of the examined regions of the genome, in contrast with previous reports [8, 10]. However, our data do suggest a minor reduction in H3K27me3 enrichment upon both firefly luciferase and the LAT enhancer during LAT-negative virus infection (Fig 4C). To functionally examine whether these reductions could be explained by the insertion of HCMV MIEP into the LAT locus rather than by an absence of LAT expression, HSV-1 microRNA and reporter gene transcription were determined from mouse TGs latently infected with previously characterised HSV-1 recombinants SC16CMVCre, SC16CMVCreΔLAT-GFP and SC16CMVCreΔLAT [12]. QRT-PCR analysis showed no significant difference in the transcription of four HSV LAT-associated microRNAs or a Cre recombinase reporter gene between the LAT-negative viruses (S3 Fig), despite the absence of a LAT-associated HCMV MIEP in recombinant SC16CMVCreΔLAT. These data suggest that the HCMV MIEP was not responsible for our observations during LAT-negative luciferase virus infection.

### The frequency of reporter gene positive neurons is increased during latency with LAT-negative HSV-1 recombinants

**Fig 5:**
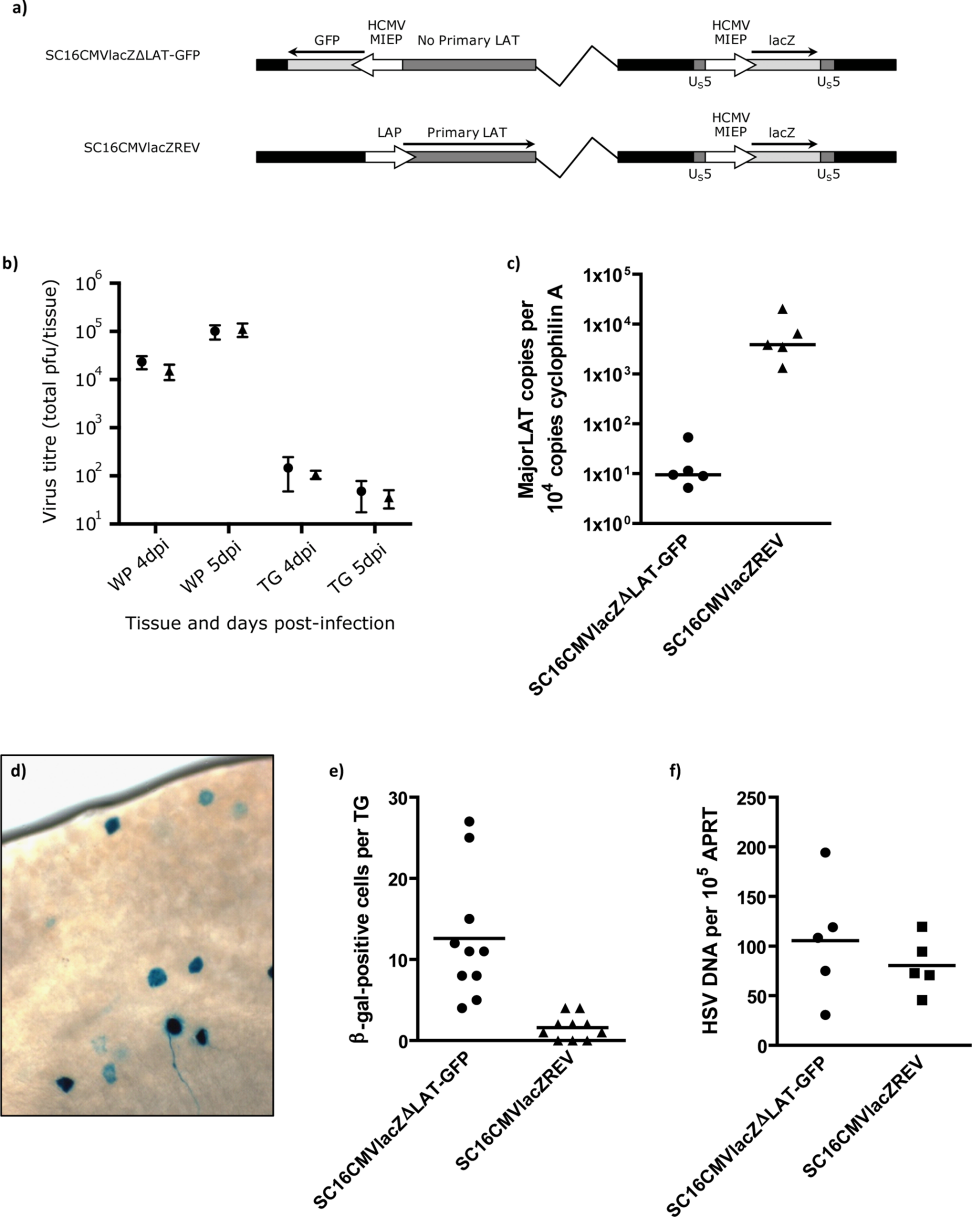
β-galactosidase reporter viruses reveal an increased frequency of gene expression in the absence of the LAT RNAs. a) Genomic structures of SC16CMVlacZΔLAT-GFP and SC16CMVlacZREV. Both viruses harbour an HCMV MIEP-lacZ expression cassette within the non-essential HSV-1 U_S_5 locus. b) Virus titres obtained from the whisker pads (WP) and trigeminal ganglia (TG) of C57BL/6, at 4 and 5 dpi. Symbols and error bars represent mean titre ± SEM from 5 mice per virus per time-point. c) LAT expression quantified by qRT-PCR from total TG pair cDNA. Each symbol represents HSV-1 major LAT intron RNA per 10^4^ copies of cellular cyclophilin A in each TG pair (N = 5 mice per virus) and floating bars represent the median of these data. Significantly less major LAT RNA was detected in SC16CMVlacZΔLAT-GFP-infected TG compared to the revertant—P = 0.0079, Mann-Whitney U test. d) Photomicrograph of β-galactosidase-positive neurons during latent infection of C57BL/6 TG. e) Quantification of β-galactosidase-positive cells during latent infection with SC16CMVlacZΔLAT-GFP and SC16CMVlacZREV. Each symbol represents the number of positive cells per TG (N = 10) and floating bars represent the mean of these data. SC16CMVlacZΔLAT-GFP-infected TG possessed significantly more positive cells than the revertant—P < 0.0005, Student's t-test. f) Virus DNA loads quantified by qPCR. HSV-1 DNA was normalised to the cellular control, APRT. Each symbol represents normalised HSV-1 DNA per TG pair (N = 5) and floating bars represent the mean of these data.

Our data demonstrate that LAT-negative HSV-1 recombinants express up to 4.9-fold greater levels of firefly luciferase relative to LAT-positive HSV-1. Whilst ChIP analyses could not show a significant difference in H3K27me3 enrichment between all four viruses, the small reduction in this repressive chromatin marker with independent LAT-negative recombinants suggests that only a minority of genomes within TG are de-repressed in the absence of LAT expression. Thus, luciferase expression may simply be greater because it occurs in a greater number of cells at any one time during LAT-negative virus infection. In order to assess the frequency of reporter gene positive neurons, mice were infected with LAT-negative (SC16CMVlacZΔLAT-GFP) and revertant (SC16CMVlacZREV) reporter viruses, which contain an HCMV MIEP *lacZ* cassette inserted into the U_S_5 locus of the HSV-1 genome (Fig 5A and S4 Fig).

The rationale for this approach was to generate viruses expressing a reporter gene from the HSV-1 genome that would allow for the identification of single cells in dissected tissues. As *lac*Z is encoded within HSV-1 its expression is therefore indicative of a de-repressed and transcriptionally active virus genome. During acute infection, both of these viruses replicated to comparable titres in C57BL/6 mice infected with 10^6^ pfu per whisker pad (Fig 5B: N = 5 mice per virus per time-point). QRT-PCR analysis of RNA from TGs of mice latently infected (30 dpi) with both viruses demonstrated a 400-fold reduction in major LAT expression from the LAT-negative mutant SC16CMVlacZΔLAT-GFP (Fig 5C: N = 5 mice per virus). During latency (32 dpi), TG were dissected, incubated in Xgal to detect *lac*Z expression from HSV-1 genomes (Fig 5D) and β-gal-positive cells enumerated. SC16CMVlacZΔLAT-GFP-infected TGs contained 7.9x as many positive cells as the SC16CMVlacZREV revertant virus (P = <0.0005, Student's t-test) with an average (± SEM) of 12.6 ± 2.5 and 1.6 ± 0.5 β-gal-positive cells per TG, respectively (Fig 5E). SC16CMVlacZΔLAT-GFP-infected mouse TG contained 1.3x as much HSV-1 DNA as SC16CMVlacZREV (Fig 5F). This difference was not significant (P = 0.43; Student's t-test) and cannot account for the 7.9x increase in β-gal-positive cells in the absence of LAT expression during latency. An increased frequency of β-gal-positive cells during SC16CMVlacZΔLAT-GFP infection was also determined relative to a wildtype HSV-1 recombinant harbouring the same HCMV MIEP-lacZ expression cassette (S4C Fig). Whilst it is possible that post-transcriptional down-regulation of *lacZ* expression by the LAT intron or LAT microRNAs may be responsible for these data, taken together with our observations during luciferase virus infection, we interpret these data to suggest that in the absence of LAT expression, genome de-repression occurs at a higher frequency than wildtype virus.

### Reporter gene expression decreases with time and is most pronounced during LAT-negative HSV-1 latency

**Fig 6:**
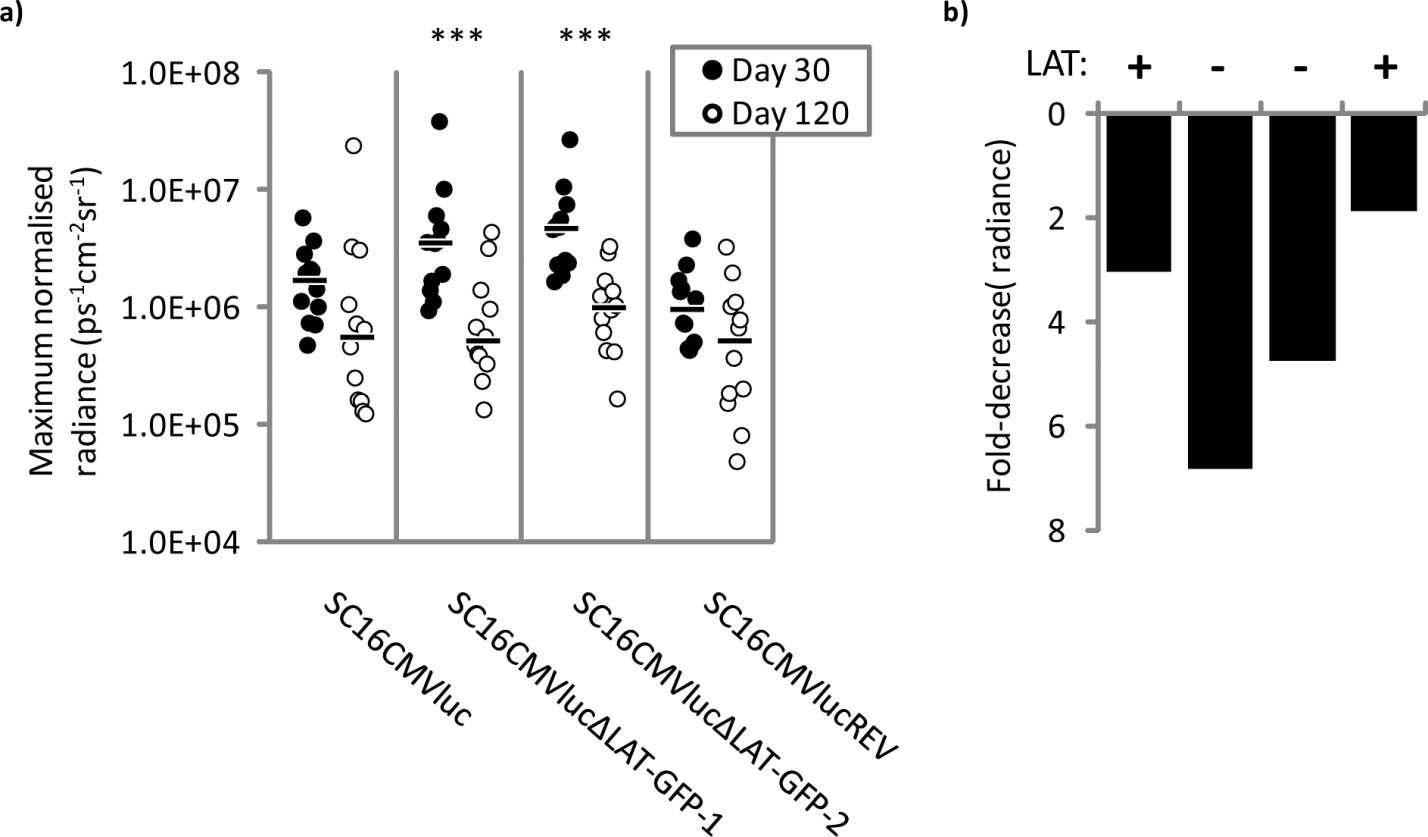
Luciferase expression decreases following long-term latent infection. a) Luciferase signal was quantified with Living Image software and normalised to relative HSV-1 DNA loads within the same TGs. Data acquisition was performed separately on 30 and 120 dpi. Each symbol represents normalised luciferase signal from an individual TG. Floating bars represent the median signal of each virus group. A signal of 1x10^4^ ps^-1^cm^-2^sr^-1^ represents a background threshold of detection. A significant reduction in luciferase signal was observed for both LAT-negative viruses (P < 0.01). b) The fold-decrease in median luciferase signal between day 30 and 120 post-infection for SC16CMVluc, SC16CMVlucΔLAT-GFP-1, SC16CMVlucΔLAT-GFP-2 and SC16CMVlucREV, respectively. A formal statistical comparison between viruses is not possible from these data.

We have previously reported that infected cell numbers decrease during long-term latent infection with LAT-negative HSV-1 recombinants [12]. We hypothesised that this loss of cells may be a consequence of reactivating neurons, and would thus include the minority population undergoing viral transcription during latency. If so, we would anticipate that viral gene expression would decrease with time in the absence of LAT expression. To test this possibility, we assessed normalised luciferase signal from latent TGs dissected 120 dpi. These TGs were dissected from 24 C57BL/6 mice infected in parallel with animals used for 30 dpi luciferase analysis (Fig 3). Comparison between luciferase signals obtained at days 30 and 120 post-infection revealed a decrease in signal across both LAT-positive and LAT-negative virus infections ranging from 1.9–6.8-fold (Fig 6A and 6B). Decreases in luciferase signal between 30 and 120 dpi were both significant and most pronounced during LAT-negative infection (Fig 6A and 6B). Whilst these data are consistent with previous work describing decreasing reporter gene expression during latent infection from both HSV-1 promoters and heterologous promoters such as the HCMV MIEP [15, 18–20], the greater loss of luciferase expression during LAT-negative virus infection may indicate: a) that neurons harbouring transcriptionally active HSV-1 are destroyed, or conversely b) that LAT expression may aid the maintenance of gene expression in SC16CMVluc and SC16CMVlucREV viruses over time.

### LAT-negative HSV-1 recombinants reactivate more frequently during e*x vivo* culture of individual neurons

**Fig 7:**
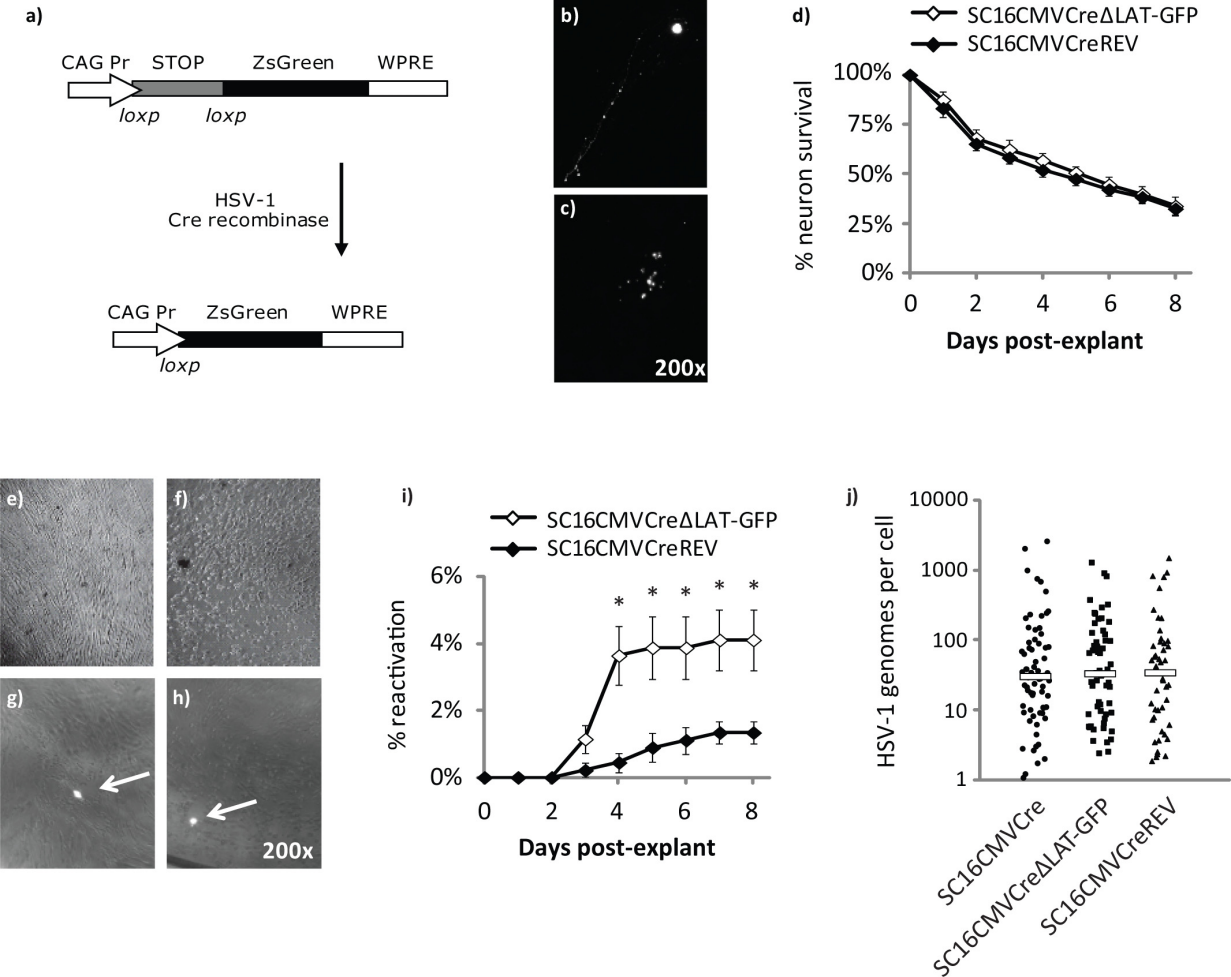
Analysis of latency at the single cell level reveals an increased frequency of reactivation during infection with LAT-negative HSV-1. a) Genomic structures of the Ai6 ZsGreen transgenic locus before and after HSV-1 Cre-mediated excision of the lox-STOP-lox cassette. The constitutively active CAG promoter drives expression of the locus. Following excision of the lox-STOP-lox cassette, ZsGreen mRNA is stabilised by the Woodchuck Hepatitis Virus Post-transcriptional Regulatory Element (WPRE). b) Fluorescence photomicrograph of an intact latently infected Ai6 neuron in MRC5 culture. c) Fluorescence photomicrograph of a dying latently infected Ai6 neuron in MRC5 culture. d) Neuron fragmentation was recorded each day during an eight day culture on MRC5 cells as an indicator of cell survival in culture. e) Photomicrograph of uninfected MRC5. f) Photomicrograph of MRC5 displaying HSV-1 cytopathic effect. g,h) Fluorescence photomicrographs of latently infected neurons undergoing reactivation. MRC5 CPE is visible in close proximity to the cultured neurons (arrows). i) Cumulative reactivation observed from *ex vivo* cultures of neurons latently infected with SC16CMVCreΔLAT-GFP and revertant virus, as assessed by CPE in the MRC5 feeder layer (n = 6 mice per virus). Symbols represent average percentage reactivation per day and error bars represent ± SEM. * represents P < 0.05; Student's T-test. j) Single cell HSV-1 DNA loads were distributed equivalently between LAT-positive and LAT-negative virus recombinants. Each symbol represents the HSV-1 genome copy in an individual fluorescent neuron. Floating bars represent median copies per cell in each virus group.

After using the aforementioned reporter gene experiments to study the very earliest stages of HSV-1 exit from latency, it was pertinent to assess whether our conclusions could be corroborated in studies of full reactivation and virus production. In order to assess the frequency of reactivation at the single cell level, we utilised an Ai6 ZsGreen reporter mouse [21] model of infection that allows for the marking and isolation of individual latency infected cells [22]. Following infection with Cre recombinase-expressing HSV-1 recombinants, excision of a lox-stop-lox cassette within infected cells leads to the permanent expression of ZsGreen fluorescence protein, allowing for visualisation of the latent cell reservoir (Fig 7A). All cells marked by ZsGreen fluorescence contain latent HSV-1 DNA [22].

Ai6 reporter mice were infected with 5x10^6^ pfu per whisker pad with the aforementioned LAT-negative recombinant virus SC16CMVCreΔLAT-GFP or its revertant, SC16CMVCreREV, which express Cre recombinase from the HCMV MIEP within the non-essential U_S_5 locus [12]. Between 28–30 dpi, three mice were killed per virus and TGs were dissected. TGs were dissociated into single cell suspensions as described in Materials and Methods. Individual fluorescent cells (n = 226–227) were removed from the cell suspension, placed on to MRC5 cell monolayers and monitored for virus reactivation over 8 days. Visual assessment of neuron cell body fragmentation (Fig 7B and 7C) revealed a steady loss of viable cells over the duration of the experiment, with just 32.3% and 30.4% fluorescent neurons remaining eight days post-explant for SC16CMVCreΔLAT-GFP and SC16CMVCreREV, respectively (Fig 7D). As well as providing a feeder cell layer for neuron adhesion, MRC5 cells are permissive to HSV-1 replication and allow for the detection of reactivating virus by visual assessment of CPE (Fig 7E–7H). One day post-explant, cells were heat-shocked by culturing at 43°C for 2 hours to stimulate reactivation [23]. Reactivation events were rare, with 4.1% and 1.3% of wells reactivating from SC16CMVCreΔLAT-GFP and SC16CMVCreREV-infected neurons, respectively (Fig 7I). In a separate experiment, neurons latently infected with SC16CMVCre and SC16CMVCreΔLAT-GFP were cultured in the absence of a heat-shock reactivation stimulus. Low rates of reactivation were again observed over an eight day period, with 6.1% and 3.0% reactivation by day eight for SC16CMVCreΔLAT-GFP and SC16CMVCre, respectively (S5 Fig). These data demonstrate that, at the level of individual neurons, reactivation is both limited to a minority of cells and occurs at roughly twice the frequency when the cell population is infected with LAT-negative HSV-1. To ascertain whether these increased reactivation kinetics could be attributed to increased HSV-1 DNA loads within cells infected with LAT-negative virus, TG from Ai6 mice were dissected 30 dpi and dissociated to cell suspensions. Individual fluorescent neurons were picked for single cell qPCR (n = 47–61 cells per virus group). The distribution of HSV DNA copies per cell was highly similar between SC16CMVCre, SC16CMVCreΔLAT-GFP and SC16CMVCreREV-infected neurons (Fig 7J), suggesting that the observed reactivation phenotype was solely due to the absence of LAT expression during latency in Ai6 mice.

In summary, these *ex vivo* data, in conjunction with our observations from independent reporter virus analyses of whole infected ganglia, indicate that LAT expression aids the suppression of HSV-1 reactivation in mice by repressing gene expression from the latent virus genome.

## Discussion

In this study we have generated luciferase and β-galactosidase-expressing HSV-1 recombinants in order to examine reporter gene expression at the level of both whole ganglia and single cells. We have observed that LAT-negative virus infections express higher levels of reporter gene during latency in TG, and this is probably the result of an increased number of infected cells in which virus gene expression occurs at any one time. Furthermore, by isolating individual latently infected cells and culturing *ex vivo*, this heightened gene expression is mirrored by a detectable increase in reactivation at the single cell level. Whilst we cannot formally rule out down-regulation of both firefly luciferase and β-galactosidase reporter genes by HSV-1 microRNAs, we propose that HSV-1 LAT expression limits the rate of virus reactivation, and does so by restricting gene expression from the latent genome.

We have previously described that LAT-negative viruses establish latency in a greater number of TG cells, relative to LAT-positive virus [12]. It is reasonable to suggest this may have resulted in the greater luciferase and β-galactosidase gene expression observed in this study. However, given that the infection dose, methodology and animal system (mice in both studies were based on the C57BL/6 genetic background) are identical, LAT-negative HSV-1 would be expected to establish latency in 25% more neurons than LAT-positive virus [12]. Such a number of cells is too few to account for the 7.9-fold greater number of β-galactosidase-positive cells and 2.1–4.9-fold higher luciferase signal observed during infection with independent LAT-negative recombinants in this study. Furthermore, measurements of luciferase signal were normalised to latent HSV-1 DNA loads within matched TGs (Fig 3), confirming that differing total virus DNA loads could not alone account for our observations. Taken together, these data provide robust experimental evidence that the LAT RNAs repress gene expression from the latent HSV-1 genome.

These findings corroborate an important observation by Chen and colleagues, in which increased transcription of ICP4 and TK per virus genome (5- and 10-fold, respectively) was observed during latent infection with a LAT-deletion virus [6]. Further evidence for a repressive role of the LAT comes from studies using ChIP assays to investigate the histone PTMs associated with the latent genome. Such studies have determined that LAT expression is positively correlated with a larger enrichment of constitutive and facultative heterochromatin upon HSV-1 lytic promoters [9, 10]. Indeed, following our observations of increased luciferase expression during LAT-negative HSV-1 latency, we too sought to characterise the enrichment of trimethylated H3K27 and pan-acetylated H3, markers of facultative heterochromatin and euchromatin, respectively. During latent infection with all four luciferase reporter viruses we determined that the vast majority of latent genomes were under strong epigenetic repression. Latent genomes were associated with minimal H3ac (S2 Fig) and were highly enriched with H3K27me3 (Fig 4C). This was unexpected for the LAT enhancer sequence due to its previous characterisation as a hyperacetylated region in both LAT-positive and LAT-negative viruses [24]. However, as our qPCR amplified sequence upstream of that described by Kubat and colleagues, this discrepancy could be reconciled if acetylated H3 enrichment is highly non-uniform across the entire enhancer. The reciprocal enrichment in H3K27me3 supports that the region under study was highly associated with facultative heterochromatin. Overall, the observation that the latent HSV-1 genomes were highly repressed was unsurprising, as reporter gene analysis with β-galactosidase-expressing viruses determined that only a small minority of infected neurons in each TG were positive for detectable levels of gene expression: roughly 2–13 cells (Fig 5) in an infected cell population estimated to number 500–800 latently infected cells per TG [12]. Whilst it is possible that β-galactosidase expression could have occurred in all infected neurons, but only to sufficiently high levels to detect in a small population of cells (therefore leading to an underestimation of the frequency of de-repressed HSV-1 DNA), our inability to detect acetylated H3 enrichment on the firefly luciferase genes inserted at HSV-1 U_S_5 (the same locus as found in the *lacZ* reporter viruses) does not support such a view. Therefore, following inoculation and primary infection in the mouse whisker pads, it is likely that the majority of latently infected cells contain tightly repressed genomes, with viral lytic (or reporter gene) transcription occurring in just a small number of neurons at any one time. Despite this, our inability to determine a significant difference in heterochromatin enrichment between LAT-positive and LAT-negative luciferase viruses conflicts with previous reports [7–10]. Whilst different HSV-1 strains have been suggested as a source of variation in ChIP results between research groups [8], if the LAT truly confers HSV-1 with the ability to regulate chromatin modifications, it would seem probable that a conservation of function should exist between strains. Of likely greater importance is the inconsistency between infection methods–namely the route of virus inoculation (whisker pad, corneal and footpad scarification) and the site of latency (TG vs dorsal root ganglia, DRG). For example, we and others have previously shown that LAT expression influences latency establishment in TG but not DRG [12, 25], which suggests an anatomical dependence of LAT function. Furthermore, as corneal scarification has been reported to infect nearly 30% of TG neurons with a number of HSV-1 strains [26], whisker pad scarification appears to lead to a more restricted establishment of latency, with ~500 neurons latently infected [12] out of approximately 20,000 [27] (~2.5%).

Despite this, we did observe less H3K27me3 enrichment upon both the LAT enhancer and 5’ CDS of the firefly luciferase gene of LAT-negative HSV-1 recombinants during latency (Fig 4C). Whilst these data were not determined as statistically significant, such a decrease in heterochromatin enrichment was expected due to the increased luciferase activity observed during LAT-negative infection. Whilst differences in H3K27me3 enrichment at the LAT enhancer could be influenced by the neighbouring HCMV MIEP, microRNA expression within and adjacent to the LAT was not significantly different between LAT-negative viruses with or without insertion of the MIEP (S3 Fig), suggesting no effect on neighbouring gene regulation occurred as a result of the exogenous promoter. The equivalent level of reporter gene expression from the U_S_5 locus observed from both LAT-negative viruses in the same experiment also suggests no long-range effect of the HCMV MIEP located in the LAT locus (S3E Fig).

Our observations that all four viruses were similarly highly repressed, as well as the detection of few β-galactosidase-positive cells within latent TGs, suggest that LAT-mediated epigenetic regulation does not occur uniformly across HSV-1 cell reservoirs. It is likely that our ChIP assay was simply not sensitive enough to detect genome de-repression from pooled TG samples. If reporter gene expression were only able to occur in 0.3–2.2% of the infected cell population (e.g. 2–13 cells among 500–600), the remaining transcriptionally silent 97.8–99.7% of the population would most heavily influence the outcome of our ChIP analyses. Future analysis of LAT function in single cells will likely provide more conclusive data to support the extent and mechanism by which LAT enhances HSV-1 genome silencing. Whilst we have been largely unable to determine an effect of LAT expression upon H3K27me3 enrichment, in total our data support a repressive role for the LAT during latency. Furthermore, small non-coding- and microRNAs processed from, or adjacent to, the LAT locus appear to actively target lytic gene expression [3, 4, 28, 29]. We believe that, on balance, the collected evidence suggests that at least one function of LAT transcription is to repress virus lytic gene expression.

Results from our single cell reactivation analyses also present further evidence that the LAT RNAs possess an inhibitory role during HSV-1 latency. It is widely reported that LAT-negative HSV-1 mutants possess a reactivation deficit [30–32]. Other studies have reported not only that LAT impacts upon the number of cells in which latency is established [12, 33, 34], but that the number of neurons in which latency is established directly correlates with reactivation [35]. Within our study we sought to assess reactivation from a known number of independent neurons cultured *ex vivo*. To do this, we infected Ai6 ZsGreen transgenic mice with Cre recombinase-expressing reporter viruses to permanently mark latently infected cells within the mouse TG. By dissociating ganglia and picking individual cells, we were able to culture neurons independently from the remaining ganglion tissue and assess reactivation frequency from single neurons. During these experiments we observed a higher reactivation frequency from neurons infected with LAT-negative HSV-1, relative to wildtype and revertant viruses (Fig 7 and S5 Fig). These data suggest that as a result of restricting HSV-1 gene expression, LAT-positive virus reactivation frequency is also reduced. Whilst these data conflict with aforementioned studies describing reactivation deficits from LAT mutants, in these reports it is not possible to determine the number of neurons in which latency was established, a factor with strong positive correlation to the efficiency of reactivation in the mouse [33, 35]. A reported deficit in latency establishment in the rabbit [34] may also explain the defective spontaneous reactivation observed with LAT-negative HSV-1 [32] in this model. In the present study, we determined that LAT-negative HSV-1 reactivated with higher frequency from a known number of single neurons, greatly reducing potential biases from latency establishment. Furthermore, from single cell PCR with individual neurons, we determined that LAT expression had no influence on the distribution of viral DNA in the latent population. It is therefore unlikely that LAT-negative HSV-1 reactivation was influenced by gross differences in viral load per neuron. The overall rate of reactivation we observed was low. One reason for this low incidence is likely to be the death of picked cells in culture. Indeed, over an eight-day duration, roughly 70% of neuronal cell bodies had fragmented. We did not observe reactivation from any neuron after it was recorded as dead. Previous studies assessing reactivation at the single cell level have also reported a low incidence of recurrence. For example, an average of 2.3 HSV-1 antigen-positive neurons per TG were detected 22 hours post-reactivation stimuli both *in vivo* and *ex vivo* [36], and just 23 infectious centres were observed following superinfection of wildtype TG cultures *ex vivo* [37]. In the present study, roughly 70 individual neurons were picked from a single cell suspension from each dissected mouse. Qualitatively, this did not comprise the majority of the fluorescently labelled cell reservoir, and thus the number of neurons observed to reactivate was likely lower than if we had analysed every infected cell within each TG pair. Spontaneous reactivation within the mouse model is yet more infrequent [12, 38] and thus it is likely that a small pool of reactivation-competent cells exists within the mouse TG. However, it remains unclear why reactivation occurs in one neuron yet latency persists in another. Clearly, the inability to express the LAT does not guarantee that reactivation will occur, as 93–96% of our neurons did not reactivate. Indeed, multiple factors are thought to be required for full reactivation, including *de novo* synthesis of VP16 [39], as well as VP16 and/or HCF-1 relocalization from the cytoplasm to the nucleus [40, 41]. Using an elegant fluorescent in situ hybridisation approach, it has been determined that LAT transcription most often occurs within neurons infected with multiple copies of the HSV-1 genome, and is negatively regulated by association with promyelocytic leukemia (PML) nuclear bodies and centromeres [42]. Whilst these observations suggest that neurons containing few copies of the HSV-1 genome may be sufficiently repressed by cellular factors, experiments from our laboratory show that reactivation can occur from neurons harbouring low copy numbers (despite occurring at an increased frequency when latent HSV-1 genomes are more numerous) [22].These data are in agreement with the observation that the reactivation competence of HSV-1 strains positively correlates with the average viral genome copy within individual neurons [26].

We have previously reported that infected cell numbers are less stably maintained in the absence of LAT expression [12]. In the current study we hypothesised that this loss may occur in populations of neurons possessing transcriptionally active HSV-1 genomes. Indeed, when we compared luciferase expression between 30 and 120 days, we did observe a significant decrease in reporter gene activity during LAT-negative infection, with a smaller reduction also observed during LAT-positive virus infection (Fig 6). Whilst these observations support our hypothesis, they do not rule out the possibility that LAT expression could have prolonged the long-term expression of firefly luciferase, thus decreasing the relative loss of signal observed from viruses SC16CMVluc and SC16CMVlucREV. Such an interpretation is supported by reports describing decreased lytic gene transcription during latency from a LAT promoter deletion mutant in rabbits [7], as well as an enhanced enrichment of the same mutant with facultative heterochromatin marker H3K27me3 relative to wildtype HSV-1 [8]. However, we interpret the sum of our data to support the LAT as a general repressor of gene expression, in agreement with other analyses of latent gene expression [2, 6] and PTM of chromatin upon the HSV genome [9, 10]. Given this interpretation, we currently favour the explanation that we are observing a loss of infected cells with time.

Whilst we are unable to dismiss the possibility that such a loss of luciferase signal would not be due to the mouse immune system targeting firefly luciferase- or GFP-positive cells for destruction, we have previously observed a reduction in latently infected cell numbers with both GFP-positive and GFP-negative LAT-deficient viruses [12], suggesting that cell death would not solely be due to reporter gene recognition by the immune system. Therefore, we believe that whilst transcriptionally permissive cells are those that are lost during infection, this is either a result of cytolytic reactivation or immune detection of viral proteins, and is exacerbated by an absence of LAT transcription in those cells.

In summary, our studies suggest that the HSV-1 latency-associated transcript reduces the frequency of reactivation at the single cell level, and do so by globally repressing virus gene expression. We have also provided further evidence that LAT expression leads to a more stable maintenance of latency throughout long-term infection, which may serve to increase the potential for transmission of the virus throughout the life of the host.

## Materials and Methods

### Cells and viruses

All viruses were derived from HSV-1 strain SC16 [43]. Baby hamster kidney (BHK) cells (American Type Culture Collection CCL-10) were used for virus stock production and plaque assays and were maintained in Dulbecco's modified Eagle's medium (DMEM) containing 10% foetal calf serum (FCS), 10% tryptose phosphate broth, 2 mM L-glutamine, penicillin (100 U/ml), and streptomycin (100 μg/ml) (PAA laboratories). MRC5 cells (American Type Culture Collection CCL-171) were used for reactivation experiments and were maintained in Dulbecco's modified Eagle's medium (DMEM) containing 10% foetal calf serum (FCS), 2 mM L-glutamine, penicillin (100 U/ml), streptomycin (100 μg/ml) (PAA laboratories) and supplemented with non-essential amino acids (Gibco). All cells were incubated at 37°C and 5% CO_2_.

### Plasmids

All HSV-1 genetic coordinates used throughout this study were determined from GenBank accession number NC_001806 [44].

pHD5-CMVluc contains a 2,833 bp fragment consisting of the human cytomegalovirus (HCMV) major immediate early promoter (MIEP) of pcDNA3 (Invitrogen) adjacent to the firefly luciferase and polyA signal from pGL4.10[luc2] (Promega), inserted into the synthetic polylinker of plasmid pHD5 [15], inserting the expression cassette at HSV-1 nt 137,945 in the Unique-Short 5 (U_S_5) open reading frame.

pPSTD1 [45] comprises a 3.3 kb *Hpa*I fragment of HSV-1 strain SC16 (HSV-1 nucleotides [nt] 117010 to 120301), containing the HSV-1 LAT promoter.

pPSTDΔLAT-CMVGFP [12] was derived from pPSTD1 and contains a 1,369-bp NsiI-PstI fragment derived from pEGFP-C1 (Clontech) encoding green fluorescent protein (GFP) under the control of the HCMV MIEP in place of the 203 bp *Pst*I LAT promoter (LAP).

pGAL1 [46] comprises the 3.8 kb HCMV MIEP lacZ-expression cassette of pMV10 [46] inserted into the HSV-1 U_S_5 open reading frame (HSV nt 137,945).

### Construction and characterization of recombinant viruses

HSV-luc has been previously characterised [14]. In this work we here term the virus SC16CMVluc with reference to the other recombinants described in this study. This virus contains the 2,833 bp firefly luciferase expression cassette from pHD5-CMVluc inserted at HSV nt 137,945.

SC16CMVlucΔLAT-GFP-1 was constructed by cotransfecting *Bam*HI-linearized pPSTDΔLAT-CMVGFP and SC16CMVluc-infected cell DNA to produce a virus in which the 203 bp LAP sequence was replaced by the HCMV MIEP GFP cassette in both Repeat-Long (R_L_) genomic regions. GFP is transcribed by the HCMV MIEP in the opposite orientation to the LAT locus.

SC16CMVlucΔLAT-GFP-2 was constructed by cotransfecting *Pst*I-linearized pHD5-CMVluc and HSV CMVCreΔLAT-GFP-infected cell DNA to introduce the 2,833 bp firefly luciferase expression cassette into the U_S_5 region (HSV nt 137,945) of a previously characterised LAT promoter-negative HSV-1 recombinant [12].

SC16CMVlucREV was constructed by cotransfecting *Bam*HI-linearized pPSTD1 and SC16CMVlucΔLAT-GFP-2-infected cell DNA to produce a GFP-negative virus restored for the core LAT promoter in both R_L_ genomic regions.

SC16CMVlacZΔLAT-GFP was constructed by cotransfecting *Xmn*I-linearized pGAL1 and HSV CMVCreΔLAT-GFP-infected cell DNA to introduce the 3.8 kb β-galactosidase expression cassette into the U_S_5 region (HSV nt 137,945) of a previously characterised LAT promoter-negative HSV-1 recombinant.

SC16CMVlacZREV was constructed by cotransfecting *Bam*HI-linearized pPSTD1 and SC16CMVlacZΔLAT-GFP-infected cell DNA to produce a β-galactosidase-expressing virus restored for the core LAT promoter in both R_L_ genomic regions.

All virus recombinants were isolated and plaque purified by limiting dilution following assessment of luciferase activity, β-galactosidase-expression or GFP-positivity/negativity where applicable.

### In vitro growth curve analyses

Growth curves were performed in BHK cell monolayers at 0.01 pfu per cell, as previously described [12]. Briefly, cells were incubated for 1 h, and extracellular virus was inactivated with citric acid solution (135 mM NaCl, 10 mM KCl, 40 mM citric acid). Infected cell monolayers were sampled at set time points over a 72-h period and stored at −70°C prior to assay.

### Mouse infection

Whisker pad infections were conducted in female C57BL/6 mice (Harlan, United Kingdom) at 8 weeks of age (unless otherwise specified), as previously described [12]. Briefly, animals were anaesthetised by isoflurane inhalation and scarified through 40μl of virus inoculum (10^6^ pfu) on both whisker pads. Whisker pad infections were also conducted in male and female Ai6 ZsGreen mice [21] at 8 weeks of age, but with inoculum titres of 5x10^6^ pfu per whisker pad. Mice were killed by rising CO_2_ asphyxiation and dissected tissues were freeze-thawed, homogenized, and freeze-thawed once more prior to assay.

### Ethics statement

All animal experiments were approved by the University of Cambridge ethical review board and by the UK Home Office under the 1986 Animal (Scientific Procedures) Act as Project Licenses 80/2205 and 70/7889.

### Enumeration of β-galactosidase-positive cells in latently infected TG

Following inoculation with β-galactosidase-expressing recombinant viruses, mice were killed by CO_2_ asphyxiation 30 dpi and TGs were dissected to complete DMEM. TGs were fixed on ice for 1.5 hours in 4% paraformaldehyde-phosphate-buffered saline and incubated in X-Gal (5-bromo-4-chloro-3-indolyl-β-d-galactopyranoside) as described previously [47]. β-galactosidase-expressing cell numbers per TG were determined by microscopy.

### Single cell reactivation assays

Ai6 ZsGreen reporter mice were infected with 5x10^6^ pfu per whisker pad. Mice were killed by CO_2_ asphyxiation 30 dpi and TGs were dissected to complete DMEM on ice. To prepare single cell suspensions, TGs were finely minced and incubated in liberase (0.7 units ml^-1^) (Roche) and DNAse I (Life Technologies) in serum-free DMEM at 37°C for one hour and were gently triturated every 20 minutes with a Gilson pipette. Neurons were layered on top of a 5-step OptiPrep gradient (Sigma) [10, 15, 20, 25 and 30% densities in DMEM supplemented with 10% FCS). Following centrifugation at 800 x *g* for 15 minutes at 10°C, neuron containing layers (the lower 60% of the gradient volume) was removed, pelleted and washed before resuspension in complete DMEM. Fluorescent latently infected neurons were observed with an Olympus IX70 inverted fluorescence microscope and were manipulated using flame-pulled Pasteur pipettes. Neurons were individually picked on to confluent MRC5 cell monolayers in 96-well plates. Enriched neurons were maintained on ice for the duration of the procedure. Plates were incubated at 37°C and 5% CO_2_ and visually assessed for cell body fragmentation and the development of CPE on a daily basis. One day post-explant, cells were heat-shocked by culturing at 43°C for 2 hours to stimulate reactivation.

### 96-well assessment of luciferase activity

To isolate luciferase-expressing HSV-1 recombinants, growth medium was removed following the onset of CPE and frozen at -70°C for later virus isolation. To each well of cells, 50 μl of luciferase lysis buffer (1% Triton-X-100, 1mM DTT, in gly-gly buffer [0.025 M glycylglycine pH 7.8, 0.015 M MgSO_4_, 4 mM EGTA]) was added, and incubated at RT for 10 minutes. 25 μl of cell lysate was assayed in an opaque 96-well luminometer plate. 92 μl of assay buffer (7.5 ml gly-gly buffer, 1.5 ml KPO_4_ buffer [1 M KH_2_PO_4_, 0.1 M K_2_HPO_4_, pH 7.8.], 1 mM DTT, 2.5 mM ATP) was added to each well and mixed. The plate was then loaded into a GloMax 96-well plate reader (Promega) and luciferase activity in each well measured before and after injection of 25 μl luciferin solution (0.2 mM luciferin (Sigma) and 0.1 mM DTT dissolved in gly-gly buffer). The same methodology was utilised to analyse luciferase expression from TGs dissected during acute infection, except that TGs from each mouse were each homogenised in 200 μl lysis buffer and 25 μl of lysate was assessed in duplicate.

### In vivo luciferase imaging

Infected mice were anesthetised with isofluorane and I.P. injected with 1.5 mg D-luciferin dissolved in 100 μl of magnesium- and calcium-free PBS. Images of live mice were acquired 15 minutes post-injection of D-luciferin for a duration of 1 minute, using an IVIS imaging cabinet and charge-coupled device camera (Caliper Life Sciences). To image latently infected TGs, mice were killed by rising CO_2_ asphyxiation 13 minutes after injection of D-luciferin and dissected TGs were imaged 3 minutes later. In total, dissected TGs were imaged 22 minutes post-injection of D-luciferin, with images captured for two minutes to increase the sensitivity of signal detection.

### Nucleic acid extraction for quantitative PCR (qPCR) and reverse transcriptase qPCR

For assessment of viral DNA loads, individual TGs were homogenized and incubated in 0.5% sodium dodecyl sulfate (SDS) and 50 μg of proteinase K/ml in TE buffer (10 mM Tris HCl, 1 mM EDTA [pH 8]) overnight at 37°C. To purify the extracted DNA, samples were phenol chloroform extracted and ethanol precipitated. For assessment of LAT expression during latency, individual TGs were homogenized in 1ml TRIzol (Life Technologies) and RNA extracted following the manufacturer's protocol. Total RNA was reverse transcribed using random primers and Superscript III reverse transcriptase (RT) (Life Technologies), alongside RT-negative control RNA.

### Chromatin immunoprecipitation (ChIP) assays

To assess the enrichment of acetylated and trimethylated histone 3 protein on latent HSV-1 genomes, for each virus TGs were pooled from 3 mice and fixed with 1% formaldehyde for 15 min at room temperate. Fixative was removed and TGs added to cold 300 μl lysis buffer (1% SDS, 10mM EDTA, 50 mM Tris-HCl, pH 8.0) containing EDTA-free protease inhibitors (Roche) and 1 mM PMSF (Roche). TGs were lysed in a glass homogenizor for two minutes and the cell lysate sonicated (Qsonica Sonicator Q700; Fisher-Scientific) at 90% amplitude (~250W/sec) for 4 ½ minutes at 4˚C. Input (INP) and IP aliquots from each preparation were snap frozen and stored at -70°C. Thawed IP samples were diluted 1:10 in dilution buffer (1% TritonX-100, 2mM EDTA, 150 mM NaCl, 20 mM Tris-Cl [pH 8.0]) containing EDTA-free protease inhibitors (Roche) and 1 mM PMSF (Roche). IP samples were pre-cleared with 100 μl protein A-Sepharose (Sigma) [in 5 ml dilution buffer containing 1 mg salmon sperm DNA ml^−1^, 1 mg BSA ml^−1^ and 0.02% sodium azide] O/N at 4˚C. IP supernatants were incubated with 20μl anti-pan-acetylated H3 polyclonal antibody [Millipore 06–599] or 10μl anti-H3K27me3 polyclonal antibody [Millipore 07–499] O/N at 4˚C. Antibody–antigen complexes were isolated with protein A–Sepharose beads O/N at 4˚C and non-specific binding removed with the following washes: 1.5 ml dilution buffer, 1.5 ml TSE (0.1% SDS, 1% TritonX-100, 2mM EDTA, 150mM NaCl), 1.5 TSE + 500 mM NaCl, 1.5 ml Buffer III (0.25 M LiCl, 1% NP40, 1% Na desoxycholate, 1 mM EDTA, 10 mM Tris-Cl [pH 8.0]) and 1.5 ml TE. Antibody-antigen complexes were eluted by incubation with 1% SDS in 0.1 M NaHCO_3_ and subsequently incubated with 0.2 M NaCl at 65 °C O/N to reverse cross-linking. DNA was purified by treatment with proteinase K followed by phenol/chloroform extraction. The DNA was isolated by ethanol precipitation following the addition of glycogen and tRNA as carrier.

### Quantitative polymerase chain reaction (qPCR)

qPCRs were conducted as previously described [7]. To determine viral loads within latent TGs, HSV-1 genomes were quantified with ICP0 promoter-specific primers and probe, and normalised to the cellular housekeeping gene adenine phosphoribosyltransferase (APRT) in duplex reactions. LAT expression was quantified with major LAT-specific primers and probe, and normalised to the cellular housekeeping gene cyclophilin A. PCR products were quantified using a Corbett Research Rotor-Gene and calculated from triplicate results from each PCR. A standard curve for each gene region was generated using dilutions of appropriate plasmids. Reaction conditions utilized were 15 min at 95°C, with 45 cycles of 15 s at 95°C and 60 s at 60°C. All primer and TaqMan probe sequences used in this study are displayed in Table 1.

**Table 1:** Primers and TaqMan probes used in this study.

Primer	Sequence (5' -3')
HSV-1 ICP0pro F	GGAAAGGCGTGGGGTATAA
HSV-1 ICP0pro R	AACGTAGGCGGGGCTTC
HSV-1 ICP0pro TM	TCGCATTTGCACCTCGGCAC
HSV-1 VP16pro F	CGGATTGGGAAACAAAGGC
HSV-1 VP16pro R	CGCACAGACGAAGACCTCA
HSV-1 VP16pro TM	ACCGAATGAACCCCTGTTGGTGCT
HSV-1 LAT enhancer F	GGCGTCGGCGACATCC
HSV-1 LAT enhancer R	CCCGAGTGTTCATCTCAGGC
HSV-1 LAT enhancer TM	AAACCCGTCTGGGGTGTTTTTCGTTCC
HSV-1 Major LAT F	CCAGGCAGTAAGACCCAAGC
HSV-1 Major LAT R	GGCCGGTGTCGCTGTAAC
HSV-1 Major LAT TM	TCCCACCCCGCCTGTGTTTTT
Firefly luc 5' CDS F	GGCCCAGCGCCATTCTA
Firefly luc 5' CDS R	ACAGCCACACCGATGAACAG
Firefly luc 5' CDS TM	CCACTCGAAGACGGGACCGCC
Mouse APRT F	GGGGCAAAACCAAAAAAGGA
Mouse APRT R	TGTGTGTGGGGCCTGAGTC
Mouse APRT TM	TGCCTAAACACAAGCATCCCTACCTCAA
Mouse upHoxa5 F	CTGTGGGACAGACGCACTGTAC
Mouse upHoxa5 R	GCTCCTTCTTCCCCCACAAC
Mouse upHoxa5 TM	AGGGCCAATTCTGGGTGCATCGC
Mouse cyclophilin F	GTCTCCTTCGAGCTGTTTGC
Mouse cyclophilin R	GAGGAACCCTTATAGCCAAATCC
Mouse cyclophilin TM	ACAAAGTTCCAAAGACAGCAGAAAACTTTC

### Statistical analyses

Analysis of virus titres was conducted using one-way analysis of variance. Analysis of luciferase signal and viral DNA loads were conducted using the Mann-Whitney U and Kruskal-Wallis tests for paired- and multiple-group analyses, respectively.

### Supporting information

Additional information for supporting experimental data can be found in Supporting Methods (S1 Methods).

## Supporting Information

S1 Fig Luciferase expression is higher during LAT-negative HSV-1 infection in latent trigeminal ganglia pairs. Following injection of D-luciferin substrate, TGs were dissected at set times from each mouse. Luciferase signal was accurately quantified with Living Image software and normalised to relative HSV-1 DNA loads within the same TGs. Each symbol represents normalised luciferase signal from a pair of TG. Floating bars represent the median signal of each virus group. A signal of 1x10^4^ ps^-1^cm^-2^sr^-1^ represents a background threshold of detection.
(TIF)

S2 Fig Pan-acetylated H3 enrichment on viral and cellular DNA. a) Fold enrichment of upHoxa5 and three virus sequences with euchromatin marker H3ac. The histogram displays the average of four independent experiments (+ SEM), each comprising groups of three pooled TGs per virus. Fold enrichment of H3ac immunoprecipitation (IP) was calculated as: [Virus sequence IP ÷ (IP + Input)] ÷ [cellular APRT IP ÷ (IP + Input)]. b) Dot plot displaying the enrichment of H3ac on the HSV-1 LAT enhancer observed from four biological repeats. Symbols represent relative enrichment of sequences from three pooled TGs per virus. Fold enrichment of H3ac IP was calculated as in a).
(TIF)

S3 Fig MicroRNA and reporter mRNA transcription in different HSV-1 LAT mutants. RNA and DNA were isolated from matched groups of male and female C57BL/6 mice TG 17dpi with HSV-1 recombinants SC16CMVCre, SC16CMVCreΔLAT and SC16CMVCreΔLAT-GFP. The latter two both carry deletion of the 203bp LAP, but only SC16CMVCreΔLAT-GFP harbours an HCMV-MIEP GFP cassette in its place. Each symbol represents normalised RNA or DNA from one pair of TG and floating bars represent the median of each group for levels of a) miR-H1-5p, b) miR-H2-3p, c) miR-H4-5p, d) miR-H6-3p, e) Cre recombinase mRNA and f) HSV-1 genomes. Fractions above each group denote the number of TG positive for the quantified nucleic acid species. No significant difference was observed between LAT-negative viruses in any assay (Kruskal-Wallis with Mann-Whitney post-tests–see S1 Dataset for details of full statistical comparison).
(TIF)

S4 Fig Generation of Β-galactosidase reporter viruses. a) Genomic structure of SC16 BE8. b) Virus titres obtained from the whisker pads and TGs of C57BL/6, 4 dpi. Each symbol represents the mean titres from five mice per virus, ±SEM. c) Quantification of β-galactosidase-positive cells during latent infection of mouse TGs with SC16 BE8 and SC16CMVlacZΔLAT-GFP. Each symbol represents the number of positive cells per ganglion and floating bars represent the mean of these data.
(TIF)

S5 Fig LAT-negative HSV-1 reactivates at an increased frequency relative to the parent recombinant, SC16CMVCre. Cumulative reactivation observed from *ex vivo* cultures of neurons latently infected with SC16CMVlacZΔLAT-GFP and parental virus, as assessed by CPE in the MRC5 feeder layer. Fractions indicate the absolute number of reactivating neurons / total neurons cultured.
(TIF)

S1 Dataset Spreadsheet of study data. (XLSX)

S1 Methods Materials and methods for supplementary qRT-PCR analyses. (DOCX)
