# Supplementary figures and images for: The HSV-1 Latency-Associated Transcript Functions to Repress Latent Phase Lytic Gene Expression and Suppress Virus Reactivation from Latently Infected Neurons
Source: PLoS Pathog. 2016 Apr 7;12(4):e1005539. doi: 10.1371/journal.ppat.1005539 (PMC4824392; doi:10.1371/journal.ppat.1005539)

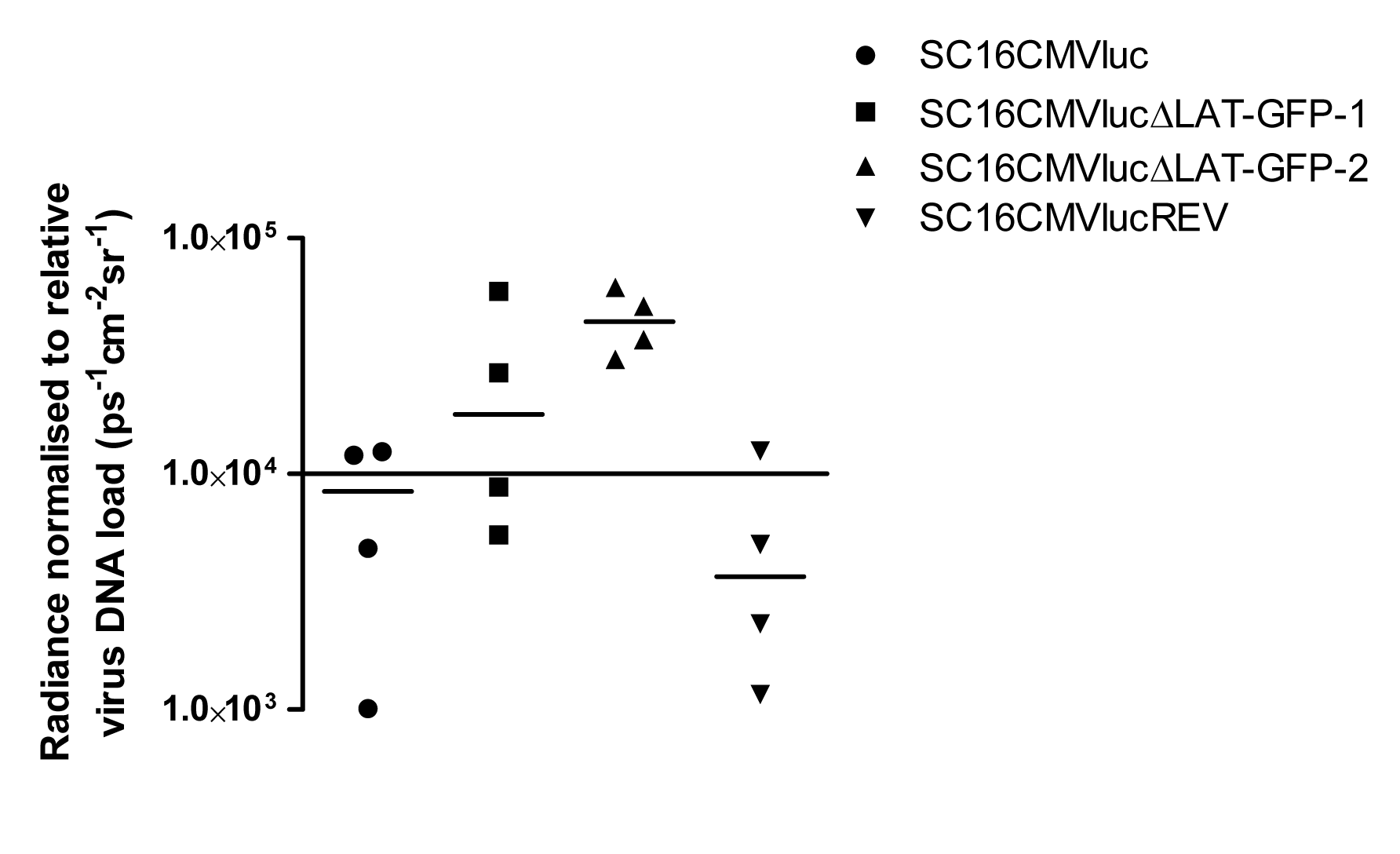

Supplement: S1 Fig — Following injection of D-luciferin substrate, TGs were dissected at set times from each mouse. Luciferase signal was accurately quantified with Living Image software and normalised to relative HSV-1 DNA loads within the same TGs. Each symbol represents normalised luciferase signal from a pair of TG. Floating bars represent the median signal of each virus group. A signal of 1x10^4^ ps-1cm-2sr-1 represents a background threshold of detection. (TIF) [file ppat.1005539.s001.tif]

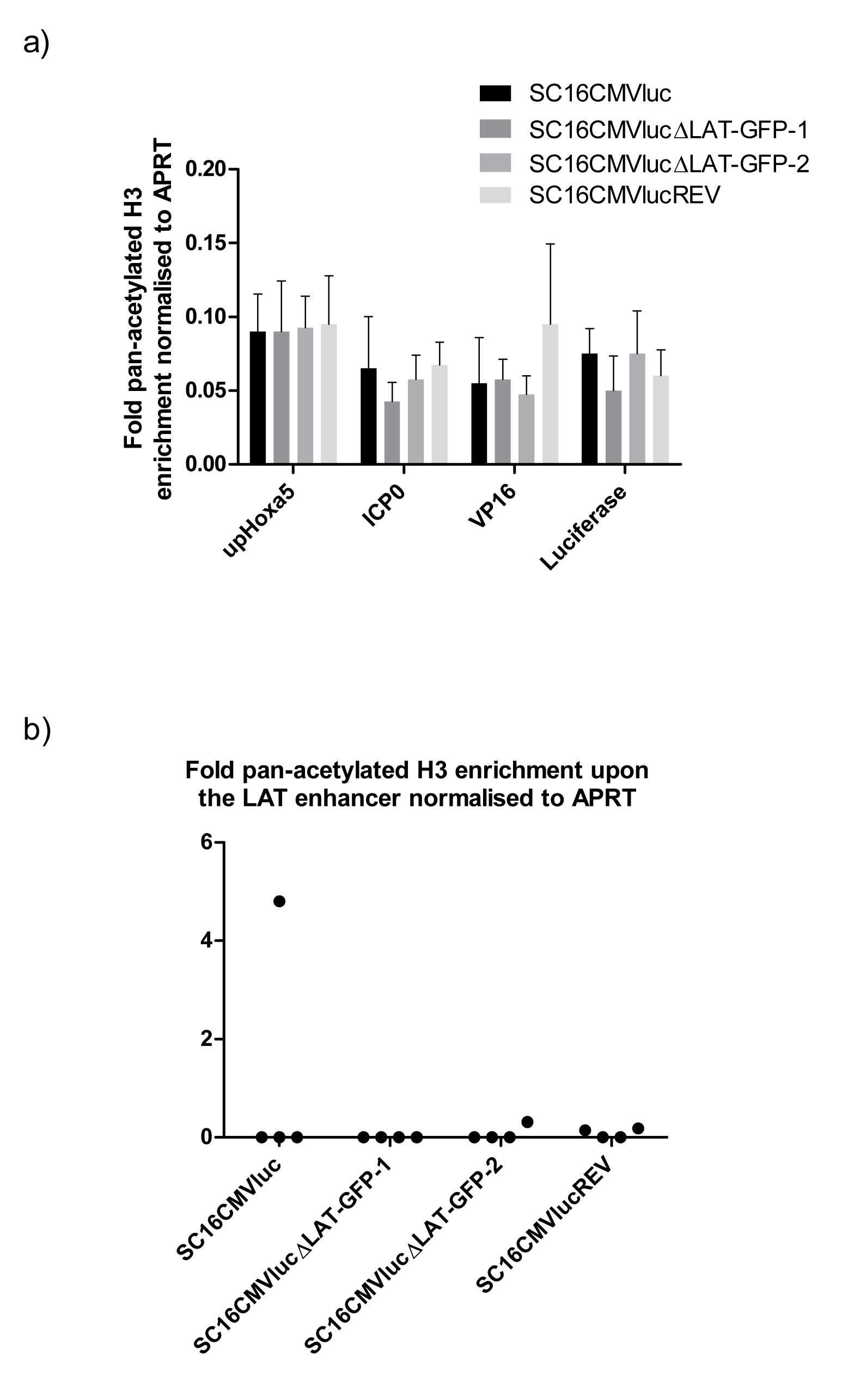

Supplement: S2 Fig — a) Fold enrichment of upHoxa5 and three virus sequences with euchromatin marker H3ac. The histogram displays the average of four independent experiments (+ SEM), each comprising groups of three pooled TGs per virus. Fold enrichment of H3ac immunoprecipitation (IP) was calculated as: [Virus sequence IP ÷ (IP + Input)] ÷ [cellular APRT IP ÷ (IP + Input)]. b) Dot plot displaying the enrichment of H3ac on the HSV-1 LAT enhancer observed from four biological repeats. Symbols represent relative enrichment of sequences from three pooled TGs per virus. Fold enrichment of H3ac IP was calculated as in a). (TIF) [file ppat.1005539.s002.tif]

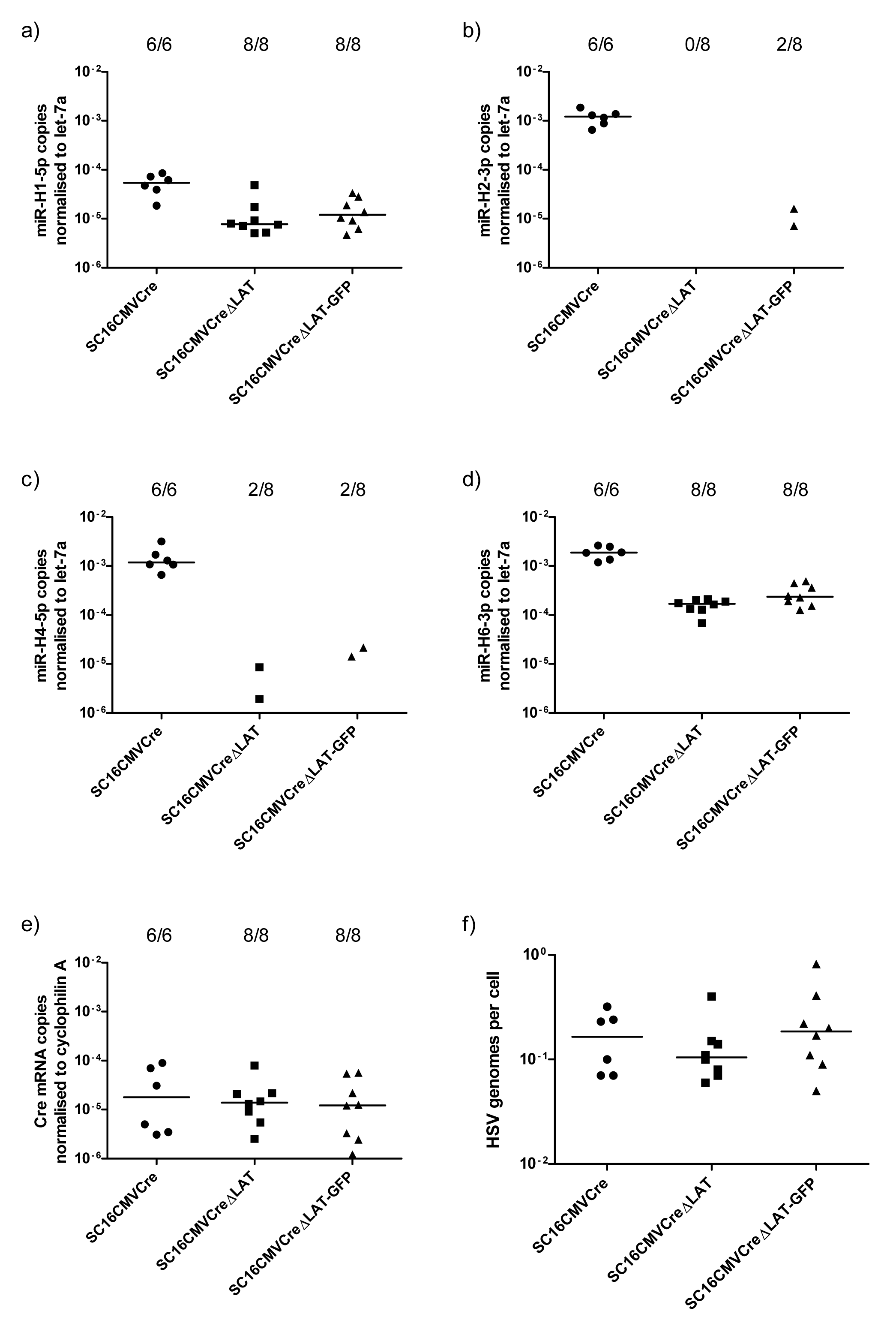

Supplement: S3 Fig — RNA and DNA were isolated from matched groups of male and female C57BL/6 mice TG 17dpi with HSV-1 recombinants SC16CMVCre, SC16CMVCreΔLAT and SC16CMVCreΔLAT-GFP. The latter two both carry deletion of the 203bp LAP, but only SC16CMVCreΔLAT-GFP harbours an HCMV-MIEP GFP cassette in its place. Each symbol represents normalised RNA or DNA from one pair of TG and floating bars represent the median of each group for levels of a) miR-H1-5p, b) miR-H2-3p, c) miR-H4-5p, d) miR-H6-3p, e) Cre recombinase mRNA and f) HSV-1 genomes. Fractions above each group denote the number of TG positive for the quantified nucleic acid species. No significant difference was observed between LAT-negative viruses in any assay (Kruskal-Wallis with Mann-Whitney post-tests–see S1 Dataset for details of full statistical comparison). (TIF) [file ppat.1005539.s003.tif]

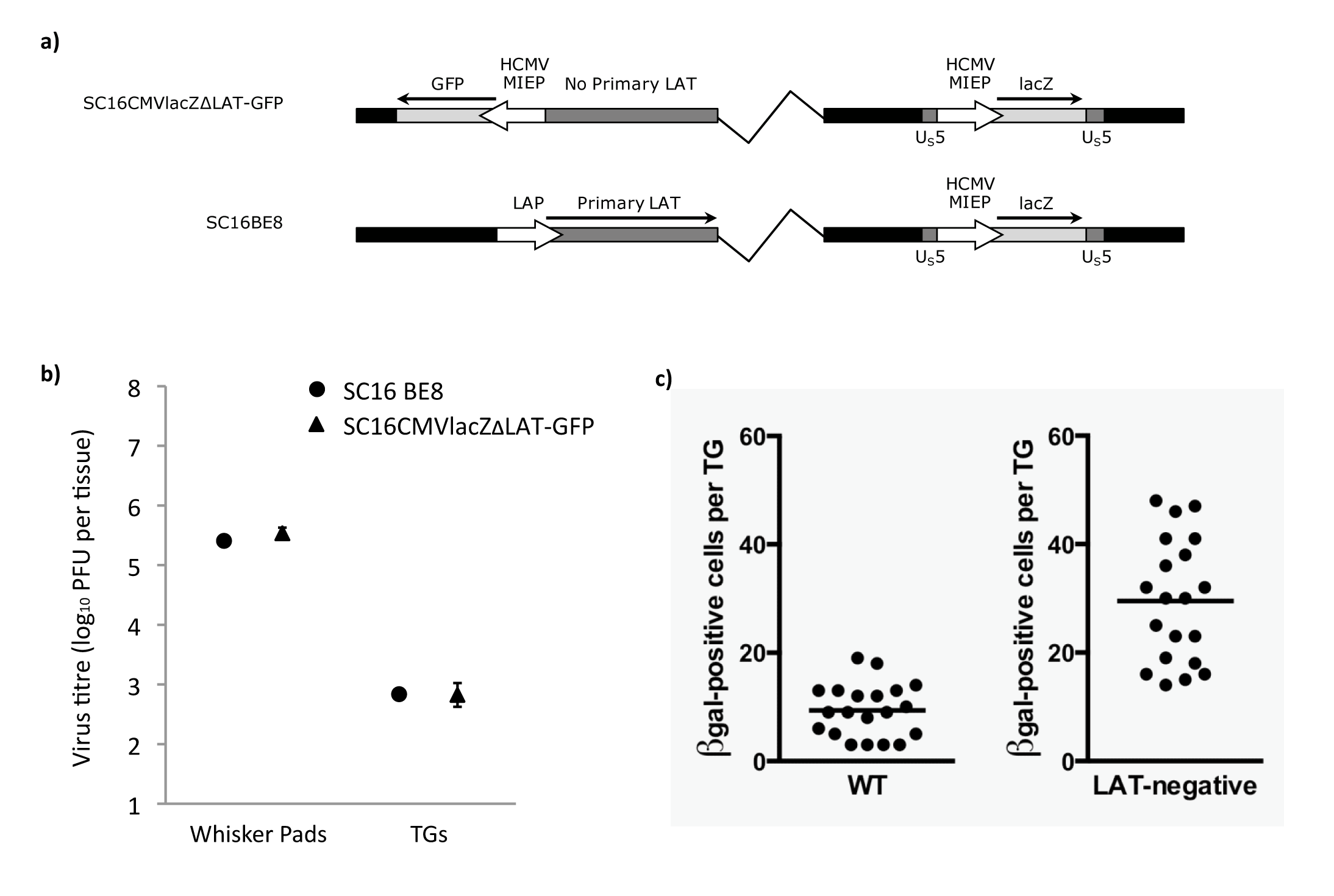

Supplement: S4 Fig — a) Genomic structure of SC16 BE8. b) Virus titres obtained from the whisker pads and TGs of C57BL/6, 4 dpi. Each symbol represents the mean titres from five mice per virus, ±SEM. c) Quantification of β-galactosidase-positive cells during latent infection of mouse TGs with SC16 BE8 and SC16CMVlacZΔLAT-GFP. Each symbol represents the number of positive cells per ganglion and floating bars represent the mean of these data. (TIF) [file ppat.1005539.s004.tif]

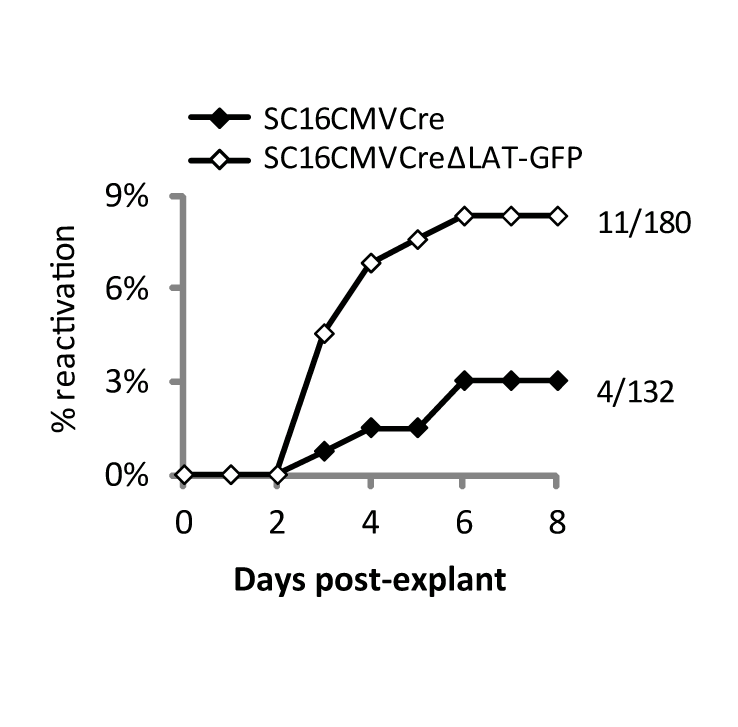

Supplement: S5 Fig — Cumulative reactivation observed from ex vivo cultures of neurons latently infected with SC16CMVlacZΔLAT-GFP and parental virus, as assessed by CPE in the MRC5 feeder layer. Fractions indicate the absolute number of reactivating neurons / total neurons cultured. (TIF) [file ppat.1005539.s005.tif]
