# Supplementary material for: The HSV-1 Latency-Associated Transcript Functions to Repress Latent Phase Lytic Gene Expression and Suppress Virus Reactivation from Latently Infected Neurons
Source: PLoS Pathog. 2016 Apr 7;12(4):e1005539. doi: 10.1371/journal.ppat.1005539 (PMC4824392; doi:10.1371/journal.ppat.1005539)
Supplement: S1 Methods — (DOCX) [file ppat.1005539.s007.docx]

**Supplementary Methods**

**Assessment of microRNA expression by TaqMan stem-loop qRT-PCR reverse transcriptase qPCR**. To assess microRNA expression, TG pairs were homogenized in 1ml TRIzol (Life Technologies) and total RNA extracted following the manufacturer's protocol. HSV-1 microRNAs H1-5p, H2-3p, H4-5p, H6-3p and cellular microRNA let-7a were reverse transcribed using a TaqMan MicroRNA Reverse Transcription Kit (ThermoFisher Scientific) with 10ng of total RNA. Stem-loop RT primers, PCR primers and TaqMan probes for each target were provided by pre-designed TaqMan microRNA assays (ThermoFisher Scientific). QRT-PCRs were conducted according to the manufacturer’s instructions.

**Isolation of DNA for qPCR from TRIzol preparations.** To assess HSV DNA loads from TG pair TRIzol (Life Technologies) homogenates, the manufacturer’s protocol was followed for the prior isolation of RNA, but modified after pelleting of the remaining interphase and organic phase material by centrifugation in 100% ethanol. Phenol traces were removed with 70% EtOH and DNA was extracted from the remaining dried pellet as described in Materials and Methods.
